# Sulindac selectively induces autophagic apoptosis of GABAergic neurons and alters motor behaviour in zebrafish

**DOI:** 10.1038/s41467-023-41114-y

**Published:** 2023-09-02

**Authors:** Wenwei Sun, Meimei Wang, Jun Zhao, Shuang Zhao, Wenchao Zhu, Xiaoting Wu, Feifei Li, Wei Liu, Zhuo Wang, Meng Gao, Yiyue Zhang, Jin Xu, Meijia Zhang, Qiang Wang, Zilong Wen, Juan Shen, Wenqing Zhang, Zhibin Huang

**Affiliations:** 1 Division of Cell, Developmental and Integrative Biology, School of Medicine, South China University of Technology, Guangzhou, 510006 China; 2 National Engineering Research Center for Tissue Restoration and Reconstruction, Key Laboratory of Biomedical Engineering of Guangdong Province, Key Laboratory of Biomedical Materials and Engineering of the Ministry of Education, Innovation Center for Tissue Restoration Reconstruction, South China University of Technology, Guangzhou, 510006 China; 3 Guangdong Provincial Key Laboratory of Pharmaceutical Bioactive Substances, Guangdong Pharmaceutical University, Guangzhou, 510006 China; 4 Division of Life Science, State Key Laboratory of Molecular Neuroscience and Center of Systems Biology and Human Health, the Hong Kong University of Science and Technology, Clear Water Bay, Kowloon, Hong Kong, People’s Republic of China; 5 Greater Bay Biomedical Innocenter, Shenzhen Bay Laboratory, Shenzhen Peking University-Hong Kong University of Science and Technology Medical Center, Shenzhen, 518055 China; 6 Greater Bay Biomedical Innocenter, Shenzhen Bay Laboratory, Shenzhen, 518055 China

**Keywords:** Toxicology, Development of the nervous system, Phenotypic screening

## Abstract

Nonsteroidal anti-inflammatory drugs compose one of the most widely used classes of medications, but the risks for early development remain controversial, especially in the nervous system. Here, we utilized zebrafish larvae to assess the potentially toxic effects of nonsteroidal anti-inflammatory drugs and found that sulindac can selectively induce apoptosis of GABAergic neurons in the brains of zebrafish larvae brains. Zebrafish larvae exhibit hyperactive behaviour after sulindac exposure. We also found that akt1 is selectively expressed in GABAergic neurons and that SC97 (an Akt1 activator) and exogenous akt1 mRNA can reverse the apoptosis caused by sulindac. Further studies showed that sulindac binds to retinoid X receptor alpha (RXRα) and induces autophagy in GABAergic neurons, leading to activation of the mitochondrial apoptotic pathway. Finally, we verified that sulindac can lead to hyperactivity and selectively induce GABAergic neuron apoptosis in mice. These findings suggest that excessive use of sulindac may lead to early neurodevelopmental toxicity and increase the risk of hyperactivity, which could be associated with damage to GABAergic neurons.

## Abstract (web summary)

Nonsteroidal anti-inflammatory drugs (NSAIDs) are widely used but their risks for early neurodevelopment remain controversial. Here, the authors showed in zebrafish larvae that sulindac induces GABAergic neuron apoptosis through autophagy activation that leads to hyperactive behavior.

## Introduction

Nonsteroidal anti-inflammatory drugs (NSAIDs), which compose one of the largest classes of drugs worldwide, are widely applied in clinical anti-inflammatory, antipyretic and analgesic treatments. NSAIDs exert their efficacy by inhibiting the activity of cyclooxygenases (COXs), affecting the metabolism of arachidonic acid, which results in the reduction of prostaglandins and other pro-inflammatory factors in vivo^1^. The most important reason for their popularity among patients is that NSAIDs can provide quick relief from headaches and toothaches; in addition, they are affordable and easy to use. According to reports, NSAIDs are currently some of the most heavily used drugs, used by more than 30 million patients every day^2,3^, but increasing side effects of NSAIDs have been reported after clinical application. Data from the USA FDA show that approximately 1/3 of all adverse drug reactions are caused by NSAIDs, with approximately 41,000 elderly people hospitalized and 3300 deaths due to NSAIDs every year^4^. For example, long-term use of NSAIDs can lead to multiple-organ side effects, including gastrointestinal toxicity, cardiovascular risk, kidney injury, hepatotoxicity, and hypertension, through COX-dependent or independent pathways^5–7^.

It should be noted that the widespread use of NSAIDs also increases the risk of acute NSAID exposure, ingestion and toxicities in pregnant women and children. For example, antenatal exposure to NSAIDs can result in a greater risk of congenital anomalies, including spina bifida^8^, premature closure of the ductus arteriosus^9^ and cardiac septal defects^10^. In children, recent studies have indicated that routine use or overdose of NSAIDs may cause life-threatening symptoms, such as upper gastrointestinal bleeding^11^, acute kidney injury^12,13^ and anaphylaxis^14^. However, only a limited number of studies have been performed to evaluate the toxicity of NSAIDs in the nervous system^15^. For example, a comparative study found that overdose of NSAIDs, particularly mefenamic acid, is associated with a dose-related risk of CNS toxicity^16^. Therefore, exploring the adverse reaction mechanism of NSAIDs, especially during neurodevelopment, will help guide clinicians to choose drugs more accurately and reasonably. We urgently need to find a convenient way to evaluate the possible toxicity of these commonly used clinical anti-inflammatory drugs in the early developmental nervous system. Zebrafish, as emerging model organisms, have been widely used in highthroughput screening and safety evaluation of drugs due to their small size, transparent body, in vitro development, ease of drug treatment and ease of observation^17^. In addition, zebrafish share considerable evolutionary similarities with mammalian species. Zebrafish genes 87% homology with human genes, and their biological structures and physiological functions are highly similar to those of mammals^18^. Moreover, the central nervous system of zebrafish is highly homologous to that of human^19^. Therefore, the zebrafish model is a good choice to evaluate the neurotoxicity of NSAIDs commonly used in clinics.

In this study, acridine orange (AO) staining was carried out on zebrafish to evaluate the effects of 46 kinds of commonly used anti-inflammatory drugs or inhibitors on neurotoxicity. We found that sulindac selectively induced apoptosis of gamma-aminobutyric acid (GABA) neurons in zebrafish and mice. Zebrafish larvae exhibited hyperactive behaviour due to the loss of these inhibitory nerves. In addition, we explored the potential mechanisms of apoptosis using the autophagy inhibitor 3-methyladenine (3-MA) and SR11237, a competitive ligand against retinoid X receptor alpha (RXRα). Our results indicated that sulindac may bind to RXRα and cause autophagy-mediated apoptosis of GABAergic neurons. Collectively, the findings of this study may provide valuable insights into the underlying mechanisms of sulindac toxicity and shed light on the risks associated with NSAIDs in early neurodevelopment.

## Results

### Sulindac elicited neurodevelopmental toxicity

**Fig. 1:**
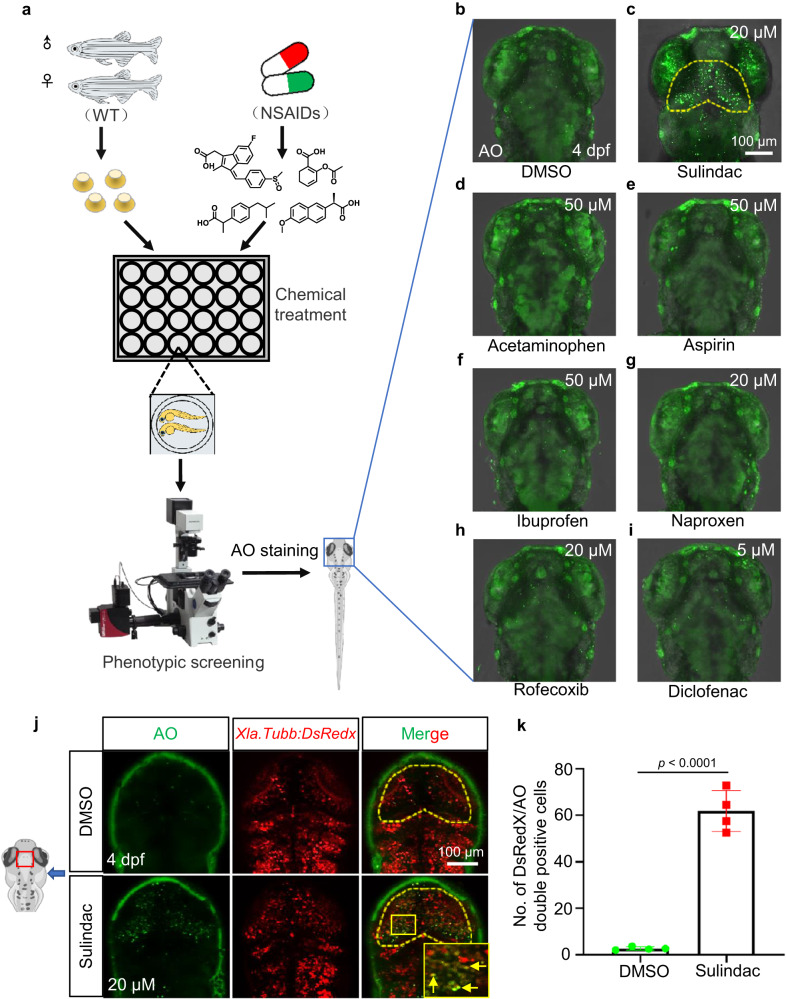
Sulindac, identified through a small clinical anti-inflammatory drug screening, elicited neurodevelopmental toxicity. **a** Flow diagram of a small-scale anti-inflammatory drug screening for brain developmental toxicity. Wild-type (WT) embryos were collected and arrayed in 24-well plates at 2 dpf. Each well contained the compound dissolved to 10 μM in DMSO and E3 embryo medium. Screening was conducted via acridine orange (AO) staining at 4 dpf. **b**–**i** Representative results of AO staining. Among the 46 anti-inflammatory drugs, only sulindac (**c**) induced apoptosis in the zebrafish midbrain (dashed line), while the other drugs, such as acetaminophen (**d**), aspirin (**e**), ibuprofen (**f**), naproxen (**g**), rofecoxib (**h**) and diclofenac (**i**), had no significant toxic effects compared to the DMSO (**b**). The experiments were independently repeated twice with similar results, and at least six zebrafish were observed each time. **j** AO+ cells (green signals) were colocalized with neuronal cells (red signals) in the midbrains of *Tg(Xla.Tubb:DsRedx)* embryos indicated by yellow arrows. **k** Quantification of the yellow double-positive cells in the midbrain. The values represent the means ± SDs (*n* = 4 larvae for per group, each dot denotes one larva). Statistics calculated by unpaired two-tailed Student’s *t* test. Source data are provided as a Source Data file.

To investigate the possible toxic effects of anti-inflammatory drugs being used in clinical treatment, we conducted a small-scale anti-inflammatory drug screening for brain developmental toxicity (Fig. 1a). Zebrafish embryos were collected, and five embryos were arrayed at 2 days post-fertilization (dpf) in each well of a 24-well plate, with the compound to be tested dissolved at 10 μM in DMSO and E3 embryo medium. The fish were then screened at 4 dpf by AO staining. To examine whether the compounds had specific effects, we retested them at varying concentrations and confirmed the zebrafish phenotypes. Finally, we screened a total of 46 NSAIDs listed in Supplementary Table 1, and only sulindac caused obvious apoptosis in the midbrains of zebrafish larvae, as indicated within the dashed lines in Fig. 1c. In contrast, DMSO (Fig. 1b, as a control group) and the other drugs, including acetaminophen (Fig. 1d), aspirin (Fig. 1e), ibuprofen (Fig. 1f), naproxen (Fig. 1g), rofecoxib (Fig. 1h) and diclofenac (Fig. 1i), had few toxic effects. Interestingly, these apoptotic cells were specifically distributed in the midbrain, while no apparent apoptotic cells were observed in the trunk region (Supplementary Fig. 1). To further determine the cell types of apoptosis in the midbrain, neuron-labelled transgenic zebrafish *Tg(Xla.Tubb:DsRedx)* and vasculature-labelled transgenic zebrafish *Tg(flk1:mCherry)* were used to colocalize apoptotic cells (Fig. 1j, k; Supplementary Fig. 2). The results showed that the vast majority of apoptotic cells were neuronal cells, suggesting that sulindac exerts adverse effects on early neurodevelopment in zebrafish larvae.

### Sulindac selectively induced apoptosis of GABAergic neurons and altered motor behaviour in zebrafish larvae

**Fig. 2:**
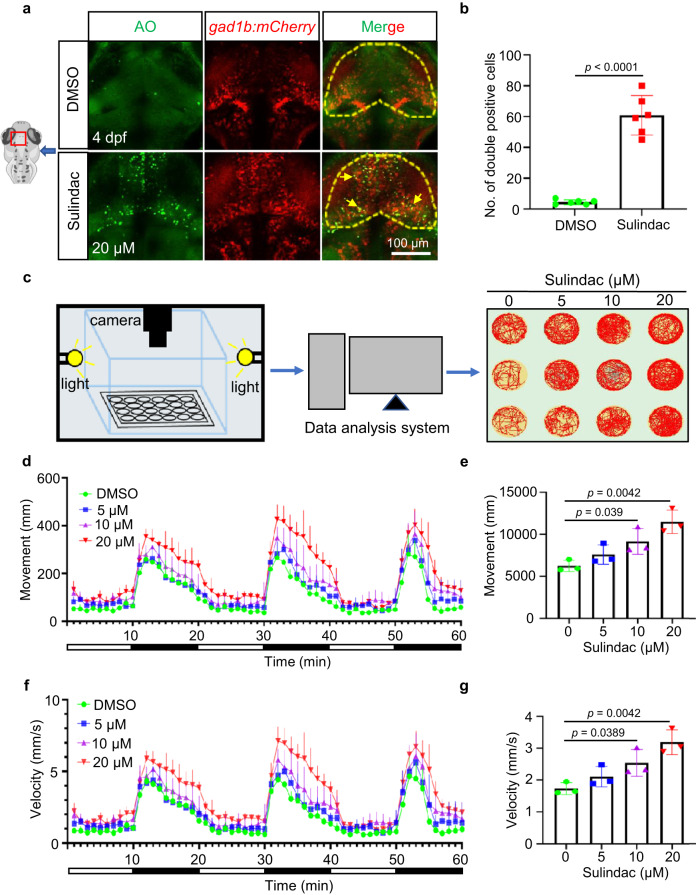
Sulindac selectively induced apoptosis of GABAergic neurons and altered motor behaviour in zebrafish larvae. **a** Most of the AO+ cells (green signals) were colocalized with mCherry+ GABAergic neurons (red signals) in the midbrains of *Tg(gad1b:mCherry)* embryos after sulindac treatment indicated by yellow arrows. **b** Quantification of the double-positive cells (yellow signals) in the midbrains of embryos. The values represent the means ± SDs (*n* = 6 larvae for per group, each dot denotes one larva). Statistics calculated by unpaired two-tailed Student’s *t* test. Source data are provided as a Source Data file. Apoptosis of GABAergic neurons in the midbrain is indicated by yellow arrows. **c** A system of behavioural experiments for recording larval motion trials in 60 min, and three representative photographs are shown for each group. **d**, **f** Swimming distance and velocity behavioural data were binned into 1-min intervals for analysis. The total movement (**e**) and average velocity (**g**) of zebrafish larvae exposed to sulindac at 5 dpf were examined during the light–dark photoperiod stimulation test (60 min). All data are presented as the means ± SDs (*n* = 3 independent biological replicates, eight larvae per replicate). Statistics calculated by unpaired two-tailed Student’s *t* test. Source data are provided as a Source Data file.

To further explore which types of neurons were affected by sulindac, different types of zebrafish neuron transgenic lines were used. In particular, apoptotic cells were colocalized with mCherry^+^ GABAergic neurons in *Tg(gad1b:mCherry)* transgenic zebrafish (Fig. 2a, b) but not with glutamatergic neurons in *Tg(vglut2a:GFP)*, cranial motor neurons in *Tg(islet:GFP)* or retinal ganglion cells in *Tg(atoh7:mCherry)* transgenic zebrafish (Supplementary Fig. 3a–c). Therefore, these results suggested that sulindac selectively promoted the apoptosis of GABAergic neurons. Interestingly, it has been shown that GABAergic circuitry is involved in motor behaviour^20^. To further confirm whether the apoptosis of GABAergic neurons induced by sulindac affected the locomotor behaviour of zebrafish larvae, a photoperiod stimulation test was used to monitor the behavioural traces of larvae at 5 dpf (Fig. 2c). Records of larval motion trails showed that compared with controls, sulindac-exposed zebrafish exhibited hyperactive behaviour, especially in the highest dose group. In addition, sulindac resulted in increases in total swimming distance (11497.72 ± 1396.99 mm) and speed (3.19 ± 0.39 mm/s) in the highest dose group in comparison to the control group (6253.65 ± 674.491 mm and 1.74 ± 0.19 mm/s, respectively) during light–dark transition stimulation (Fig. 2d–g). This behaviour showed hyperactivity, which may have been attributable to the damage to GABAergic neurons caused by sulindac.

### Sulindac selectively induced apoptosis of GABAergic neurons via the Akt pathway in zebrafish

**Fig. 3:**
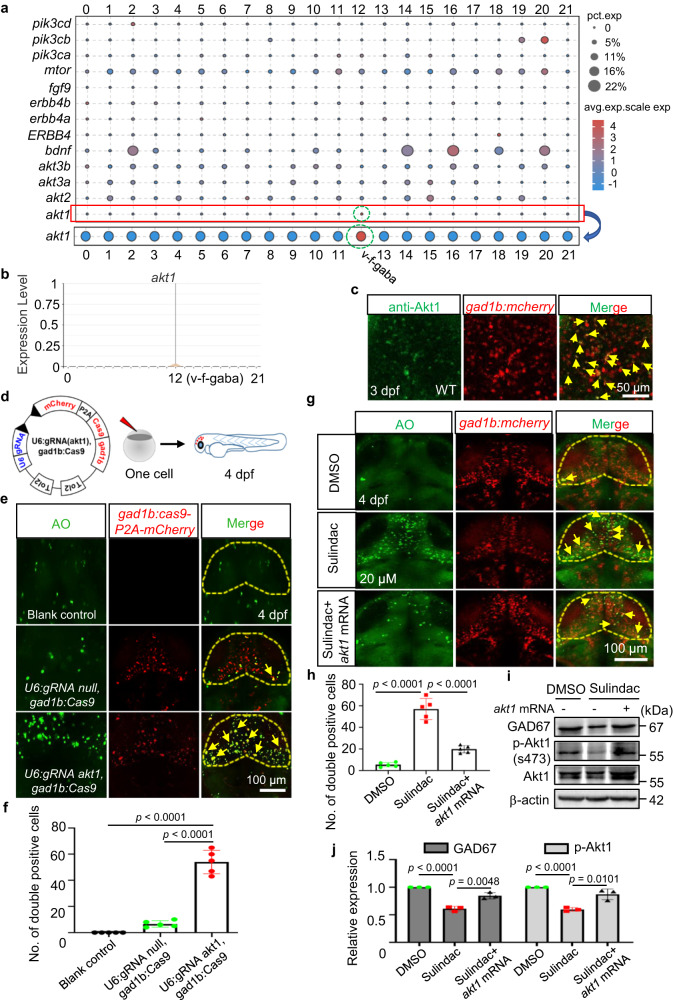
Sulindac selectively induced apoptosis of GABAergic neurons via Akt signalling in zebrafish. **a** Gene dot plot showing that only *Akt1* was abundant in GABAergic neurons, but not *akt2, akt3a, akt3b, bdnf, erbb4* (*erbb4a, erbb4b, ERBB4*)*, fgf9, mtor*, or *pi3k* (*pi3ca, pi3cb, pi3cd*). **b** Gene violin map distribution showed that *akt1* was distributed mainly in the subpopulation of GABAergic neurons. **c** Double immunostaining revealed that Akt1 proteins were selectively expressed in GABAergic neurons in the midbrains of *Tg(gad1b:mCherry)* embryos, as indicated by yellow arrows. The experiment was repeated twice with similar results, and at least five zebrafish were observed each time. **d** Flow diagram showing a tissue-specific CRISPR vector microinjected to knoc kout *akt1* in GABAergic neurons. **e** Specific knock out of *akt1* in GABAergic neurons via injection with the *gad1b:Cas9-T2A-mCherry,U6:gRNA akt1* vector led to apoptosis of GABAergic neurons, as indicated by yellow arrows. **f** Quantification of the yellow colour double-positive cells in the midbrains of embryos. The values represent the means ± SDs (*n* = 5 larvae for per group, each dot denotes one larva). Statistics calculated by unpaired two-tailed Student’s *t* test. Source data are provided as a Source Data file. **g** The number of apoptotic GABAergic neurons was restored by overexpression of *akt1* mRNA after sulindac treatment, as indicated by yellow arrows. **h** Quantification of the yellow double-positive cells in the midbrains of embryos. The values represent the means ± SDs (*n* = 5 larvae for per group, each dot denotes one larva). Statistics calculated by unpaired two-tailed Student’s *t* test. Source data are provided as a Source Data file. Apoptosis of GABAergic neurons in the midbrain is indicated by yellow arrows. (**i**) Western blot analysis showed that overexpression of *akt1* inhibited the declines in p-Akt1 and Gad67 protein levels. **j** The data are presented as the means ± SDs (*n* = 3 independent biological replicates). Statistics calculated by unpaired two-tailed Student’s *t* test. Source data are provided as a Source Data file. β-Actin was used as an internal control.

As we observed that sulindac selectively induced apoptosis of GABAergic neurons in the midbrains of zebrafish larvae above, we examined the underlying mechanism of sulindac selectivity in GABAergic neurons. To do so, we identified some genes that have been reported to be associated with GABAergic neuron development, including *akt*^21^, *bdnf*^22^, *erbb4*^23^, *fgf9*^24^, *mtor*^25^ and *pi3k*^26^. Interestingly, single-cell RNA-sequencing data from the whole brains of zebrafish larvae (GEO accession GSE212888) showed that only *akt1*, a homologue of mammalian *Akt1*, but not *akt2* and *akt3*, was specifically expressed in a subpopulation of GABAergic neurons of 3-dpf zebrafish larvae (Fig. 3a, b; Supplementary Fig. 4, and Supplementary Table 2). The expression of *akt1* did not occur in many cells but was sufficient to provide a clue regarding the underlying mechanism. However, *bdnf*, *erbb4* (*erbb4a, erbb4b, ERBB4*), *fgf9*, *mtor*, and *pi3k* (*pi3ca, pi3cb, pi3cd*) were not restricted to GABAergic neurons (Fig. 3a). Furthermore, we also found that approximately 50% of gad1b:mCherry neurons expressed Akt1 in the zebrafish midbrain by immunofluorescence staining (Fig. 3c). AKT is a kinase with three isoforms: AKT1, AKT2 and AKT3^27^. These isoforms are functionally distinct due to differences in target specificity^28,29^. Emerging evidence suggests that AKT1 is highly expressed in the mammalian brain and plays a crucial role in brain development^30,31^. Therefore, we hypothesized that Akt1 may play an important role in GABAergic neuron cell death induced by sulindac.

To further support this notion, we performed tissue-specific knockout of *akt1* in GABAergic neurons or overexpression of *akt1* to observe the apoptotic regulation of *akt1* on GABAergic neuron cell death. For tissue-specific knockout of *akt1* in GABAergic neurons, a tissue-specific CRISPR vector (*gad1b:Cas9-T2A-mCherry, U6:gRNA akt1*) was applied^32,33^, which contains two key promoters: one is the zebrafish U6 promoter, which can drive the expression of the guide RNA (gRNA) *akt1*, and the other is the zebrafish gad1b regulatory element, which tissue-specifically controls the expression of zebrafish codon-optimized Cas9 and the fluorescent reporter mCherry (Fig. 3d). To ensure the reliability of the knockout results, the knockout efficiency of the *akt1* target sequence and the CRISPR vector were verified (Supplementary Fig. 5 and Supplementary Fig. 6). Notably, AO staining showed that tissue-specific knockout of *akt1* significantly increased the apoptosis of GABAergic neurons (Fig. 3e, f), which was also consistent with the results induced by treatment with the AKT inhibitor MK-2206 (Supplementary Fig. 7). In contrast, we found that the changes in the number of apoptotic GABAergic neurons (Fig. 3g, h) and the expression of Gad67 protein following sulindac treatment (Fig. 3i, j) were able to be reversed by overexpression of *akt1* mRNA but not by overexpression of *akt2* mRNA (Supplementary Fig. 8a–e) or *akt3* mRNA (Supplementary Fig. 8a, b, f–h). Furthermore, we found that SC97, a major activator of brain-permeable Akt1 phosphorylation^34^, also significantly attenuated sulindac-induced GABAergic neuronal apoptosis (Supplementary Fig. 9). These data support the notion that sulindac may induce GABAergic neuron cell death by reducing p-Akt levels in the midbrains of zebrafish larvae.

### Sulindac induced apoptosis via the mitochondrial pathway

**Fig. 4:**
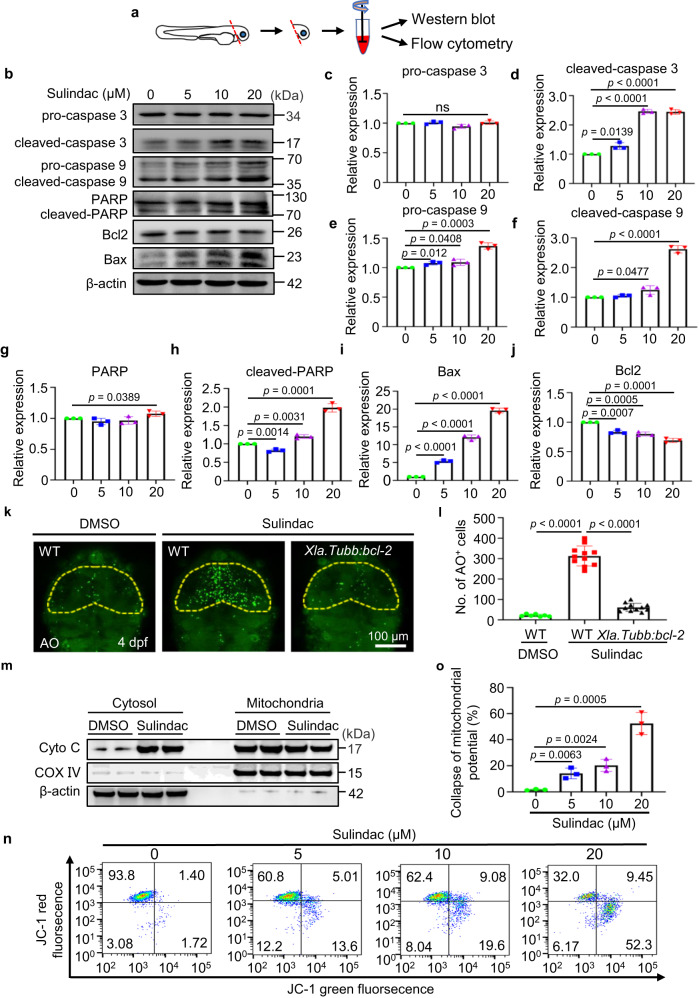
Sulindac induces apoptosis via the mitochondrial pathway. **a** Schematic diagram showing that the head tissue of embryos was split for Western blotting and flow cytometry. **b** Western blot analysis of the protein levels of apoptosis-related proteins with sulindac treatment at increasing concentrations. The immunoblot intensity was determined by ImageJ analysis software. **c**–**j** The data are presented as the means ± SDs (*n* = 3 independent biological replicates). NS, not significant. Statistics calculated by unpaired two-tailed Student’s *t* test. Source data are provided as a Source Data file. β-Actin was used as an internal control. **k** After treatment with sulindac or DMSO, the number of apoptotic neurons (AO + ) decreased significantly after overexpression of bcl2 in the midbrains of Tg(Xla.Tubb:bcl-2) embryos. **l** Quantification of AO+ cells in the midbrain. *n* = 7, 11 and 11 for the DMSO (WT) group, sulindac (WT) group, and sulindac (Xla.Tubb:bcl-2) group, respectively. Each dot in (**l**) denotes one larva. The values represent the means ± SDs. Statistics calculated by unpaired two-tailed Student’s *t* test. Source data are provided as a Source Data file. **m** Western blot analysis of the protein levels of cytochrome c in the cytosolic and mitochondrial fractions of zebrafish larval neurons. The experiment was repeated three times independently with similar results. **n** Flow cytometric analysis of mitochondrial transmembrane potential (ΔΨm) after staining with JC-1. Zebrafish neuronal cells were treated with increasing concentrations of sulindac. **o** Quantitative data of sulindac-induced collapse of mitochondrial potential. Each column represents the mean ± SDs (*n* = 3 independent biological replicates). Statistics calculated by unpaired two-tailed Student’s *t* test. Source data are provided as a Source Data file.

To further explore the potential apoptotic mechanisms, whole-head tissue was separated for detection (Fig. 4a). We screened proteins from a series of genes that have been reported to be associated with apoptosis, including p53, Bcl-2 and Bax, poly (ADP-ribose) polymerase (PARP), caspase 3, caspase 8 and caspase 9^35,36^. We found that the caspase cascade signalling pathway was activated, while p53 was not altered (Supplementary Fig. 10a, b). Cleaved caspase 3, cleaved caspase 9, cleaved PARP and Bax levels were increased in a dose-dependent manner (Fig. 4b–i), but the Bcl-2 level was decreased (Fig. 4b, j). Interestingly, we used a *Tg(Xla.Tubb:bcl-2)* transgenic line, in which zebrafish *bcl-2* mRNA is overexpressed specifically in neurons under the control of a Xenopus neuron-specific β-tubulin (*Xla.Tubb*) promoter, and Bcl-2 overexpression considerably reduced the number of apoptotic neuronal cells, which had been increased by sulindac (Fig. 4k, l). It is widely accepted that the interaction between the apoptosis-promoting factor Bax and the inhibitor of apoptosis Bcl-2 plays an important role in mitochondrial apoptosis and that Bax can directly bind to mitochondria, inducing the release of cytochrome c from mitochondria into the cytosol^37^. Consequently, the release of cytochrome c results in the activation of the caspase cascade, including caspase 9 and caspase 3, and further amplifies the apoptotic signal^38^. As expected, our results showed that cytochrome c accumulation in the cytoplasm was significantly increased in the sulindac group compared to the DMSO group (Fig. 4m).

The release of cytochrome c causes loss of the mitochondrial membrane potential (ΔΨm), which is a marker for mitochondrial dysfunction^39^. To further confirm that sulindac-induced apoptosis was mediated by the mitochondrial pathway, changes in ΔΨm were analysed with flow cytometry via detection of the fluorescent dye JC-1, which produces red fluorescence when the mitochondrial membrane potential is high and green fluorescence when the mitochondrial membrane potential is low^40^. Treatment with sulindac resulted in a marked collapse of ΔΨm in a concentration-dependent manner in zebrafish neuronal cells (Fig. 4n, o).

As sulindac is a well-known COX inhibitor, we designed specific MOs to knock down the expression of COXs (Ptgs1/Cox-1, Ptgs2a/Cox-2a and Ptgs2b/Cox-2b), but few apoptotic cells were observed after treatment with sulindac (Supplementary Fig. 11a–c), suggesting that COXs were not involved in sulindac-induced apoptosis. Prostaglandin E2 (PGE2) is the main effector prostanoid and is regulated by COXs^41^. However, the apoptotic effect of sulindac was not reversed by exposure to 16,16-dimethyl-PGE2 (dmPGE2; Supplementary Fig. 11d), a long-acting derivative of PGE2. Taken together, these results indicated that sulindac caused mitochondrial dysfunction and triggered Bcl2-dependent mitochondrial apoptotic pathways but not the COX-dependent pathway.

### Sulindac promoted RXRα-dependent autophagy-induced apoptosis through the PI3K/AKT/mTOR pathway

**Fig. 5:**
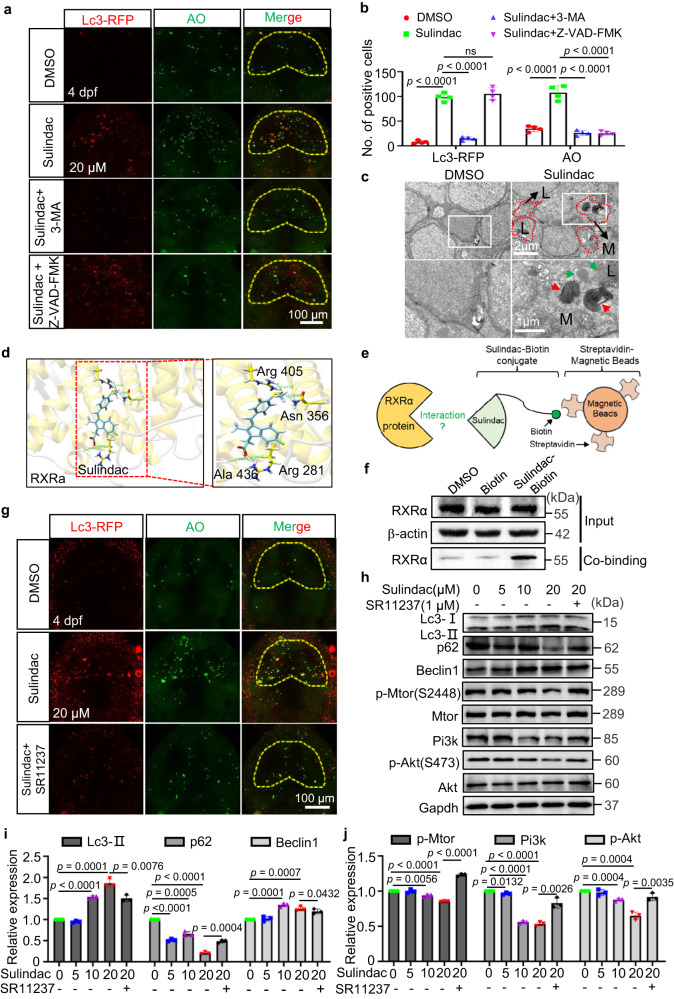
Sulindac promoted RXRa-dependent autophagy-induced apoptosis through the PI3K/AKT/mTOR pathway in zebrafish neurons. **a** Autophagy and apoptosis were evaluated according to Lc3-RFP and AO signals when treated with 3-MA (autophagy inhibitors) or Z-VAD-FMK (apoptosis inhibitors) before sulindac treatment. **b** Quantification of Lc3-RFP+ and AO+ cells in the midbrain of embryos. The values represent the means ± SDs (*n* = 4 larvae for per group, each dot denotes one larva). NS, not significant. Statistics calculated by unpaired two-tailed Student’s *t* test. **c** Transmission electron micrograph analysis of autophagy measured in the midbrains of embryos treated with sulindac or DMSO. Small autophagosomes (green arrows) and autolysosomes (dashed lines) contained degraded cellular debris (red arrows), lysosomes (L), and mitochondria (M). The experiment was repeated once with similar results, and three zebrafish were observed in each group. **d** A docking study was performed to evaluate the interaction of RXRa (PDB ID: Q90416) with sulindac (Zinc ID: 3786192) via the SwissDock server (http://www.swissdock.ch/). The most favourable binding mode showed four hydrogen bonds and had a full fitness of −2523.73 kcal/mol with −7.56 kcal/mol docked free energy; the close-up view is represented in the right panel. **e** Schematic diagram of the use of the streptavidin–biotin (SA–biotin) system to acquire proteins bound to sulindac. **f** Western blot analysis showed that there was an interaction between sulindac and RXRa protein in zebrafish head tissue. The experiment was repeated three times independently with similar results. **g** Autophagy and apoptosis were evaluated according to Lc3-RFP and AO signals upon treatment with SR11237 before sulindac treatment. The experiment was repeated twice with similar results, and at least five zebrafish were observed each time. **h** Western blot revealed the expression levels of autophagy-associated proteins and the PI3K/AKT/mTOR pathway proteins after treatment with increasing concentrations of sulindac as indicated. The immunoblot intensity was determined by ImageJ analysis software **i**, **j** The data are presented as the means ± SDs (*n* = 3 independent biological replicates). Statistics calculated by unpaired two-tailed Student’s *t* test. Source data are provided as a Source Data file. GAPDH was used as an internal control.

Previous studies have shown that both AKT and BCL-2 play a key role in modulating the interplay between autophagy and apoptosis by negatively regulating the Beclin1-dependent autophagy program^42,43^. The process of apoptosis is often accompanied by the occurrence of autophagy, which has a dual role in the process of apoptosis. Mild autophagy is a self-protecting process and is responsible for degrading damaged organelles and misfolded proteins, while excessive autophagy causes the destruction of a substantial number of normal organelles, leading to the activation of programmed cell death, called autophagic cell death^44^. To investigate the effects of sulindac on neuronal apoptosis and autophagy in zebrafish, a red fluorescent protein (RFP)-tagged Lc3 (RFP-Lc3) plasmid was injected to mark the magnitude of autophagic flux, and AO staining was used to mark apoptotic cells^45^. The results showed that both RFP-Lc3^+^ and AO^+^ cells were dramatically increased in the zebrafish midbrains after sulindac treatment (Fig. 5a, b). More interestingly, most of the RFP-Lc3^+^ cells were colocalized with AO^+^ cells. We next used electron microscopy to assess ultrastructural changes. Phenotypically, a reduced number of mitochondria and multiple small autophagosomes (Fig. 5c, green arrows), distinguished by their characteristic double membranes, were identified, as well as large autolysosomes containing electron-dense deposits (Fig. 5c, red arrows). These results further revealed that sulindac induced autophagy in neurons. Then, we explored the relationship between sulindac-induced autophagy and apoptosis by combining treatment with sulindac and 3-MA (an autophagy inhibitor) or Z-VAD-FMK (an apoptosis inhibitor). Pretreatment with 3-MA attenuated the inhibitory effect of sulindac on neuronal cell viability, but pretreatment with Z-VAD-FMK did not affect autophagy after sulindac treatment (Fig. 5a, b). These experiments demonstrated that sulindac induced Bcl-2-mediated autophagy, thereby promoting neuronal autophagic cell death.

Given the discovery of these mechanisms of sulindac, we then sought to identify the target of sulindac in activating the autophagy pathway. Here, we predicted potential targets of sulindac using the SEA Search Server database (https://sea.bkslab.org/), and the results showed that the three most correlated targets were prostaglandin G/H synthase 1 (PTGS1/COX-1), RXRα and prostaglandin G/H synthase 2 (PTGS2/COX-2; Supplementary Fig. 12). Since our data showed that sulindac induced neuronal apoptosis via a COX-independent pathway, we hypothesized that RXRα may serve as a target of sulindac to induce autophagic cell death. To test this possibility, we performed a docking study to evaluate the interaction of RXRα (PDB ID: Q90416) with sulindac (Zinc ID: 3786192) using the SwissDock server (http://www.swissdock.ch/). The results indicated that the most favourable binding modes showed four hydrogen bonds between sulindac and residues Arg405, Asn356, Arg281, and Ala436 of RXRα and a full fitness of −2523.73 kcal/mol with −7.56 kcal/mol docked free energy (Fig. 5d). It is well known that docking free energy values less than −7.0 kcal/mol indicate strong binding activity^46^. Therefore, the results demonstrated that sulindac could be tightly bound to the RXRα protein.

To further confirm the interaction between sulindac and RXRα protein in the brain tissue, sulindac was biotinylated. Assuming that sulindac interacts with RXRα, RXRα should also be indirectly biotinylated, and therefore, the RXRα protein should be captured by the SA–biotin system^47^ (Fig. 5e). As expected, the binding results showed that the amount of RXRα protein captured by sulindac–biotin was significantly increased (Fig. 5f). Together, these data imply that RXRα may serve as an intracellular target mediating the apoptotic effect of sulindac. To further investigate the role of RXRα in sulindac-induced autophagic cell death of neurons, SR11237 (a potent RXRα-selective ligand) was used^48^. Sulindac induced extensive autophagy and apoptosis but had little effect after SR11237 pretreatment of embryos (Fig. 5g). Moreover, Western blotting showed that the levels of the autophagy-related proteins LC3-II, a marker of autophagosomes, and Beclin1, which plays a key role in the formation of autophagosomes, were decreased following SR11237 treatment, while that of the p62 protein, which is involved in the degradation of autophagy, was increased sharply (Fig. 5h, i). Therefore, this result further confirmed that sulindac activated autophagy and that this process may have involved binding to the RXRα receptor.

Considering that the above studies validated that Akt1 was involved in sulindac-induced autophagic apoptosis, the PI3K/AKT/mTOR pathway, a well-known pathway in autophagy, was also examined^44^. The expression of Pi3k, p-Akt (Ser473), and p-Mtor (Ser2448) was remarkably downregulated in a dose-dependent manner, with little change in total Akt levels and Mtor levels (Fig. 5h, j). However, all these changes were reversed by SR11237. Moreover, MHY1485, an mTOR activator, significantly reversed the influence of sulindac, further supporting the notion that sulindac can arrest the PI3K/AKT/mTOR pathway, leading to the autophagic cell death of zebrafish neurons (Supplementary Fig. 13). Together, these results demonstrate that sulindac likely promotes RXRα-dependent autophagy and induces apoptosis via suppression of the PI3K/AKT/mTOR signalling pathway in zebrafish GABAergic neurons.

### Long-term administration of sulindac in mice can lead to hyperactivity and apoptosis of neocortical GABAergic neurons

**Fig. 6:**
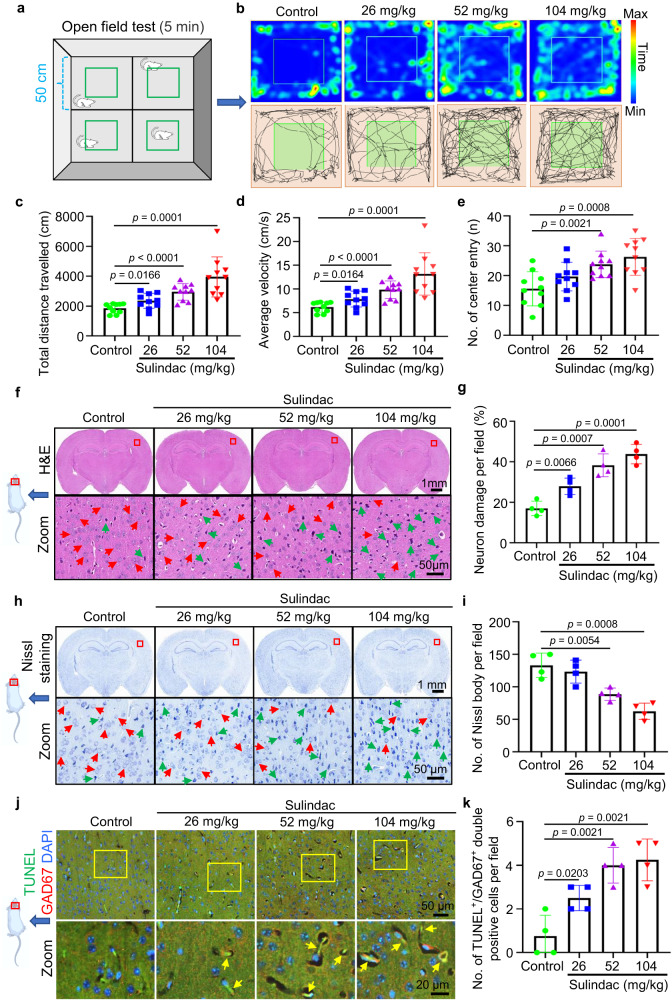
Sulindac caused mice to display hyperactivity and induced apoptosis of neocortical GABAergic neurons. **a** Schematic diagram of the monitoring the locomotion of mice in an open field. **b–e** Hyperlocomotion of mice was manifested as increases in movement distance, average speed and the number of entries into the central area after sulindac treatment. The data are presented as the means ± SDs (*n* = 10 mice per group). Statistics calculated by unpaired two-tailed Student’s *t* test. Source data are provided as a Source Data file. **f** Haematoxylin/eosin (H & E) staining showed the structural morphology of neuronal cells in the cerebral cortex of mice after sulindac treatment. Red arrows indicate normal neuronal cells, and green arrows indicate dying neurons. **g** Quantification of dying neurons in the cerebral cortex in mice. The data are presented as the means ± SDs (*n* = 4 mice per group). Statistics calculated by unpaired two-tailed Student’s *t* test. Source data are provided as a Source Data file. **h** Nissl staining demonstrated that the Nissl bodies of neurons were gradually lost in the cerebral cortex in mice in a dose-dependent manner after sulindac treatment. Normal neurons contained a large number of Nissl bodies (red arrow), which disappeared in dying neurons (green arrow). **i** Quantification of Nissl bodies in the cerebral cortex in mice. The data are presented as the means ± SDs (*n* = 4 mice per group). Statistics calculated by unpaired two-tailed Student’s *t* test. Source data are provided as a Source Data file. **j** Double staining of TUNEL/GAD67 in the cerebral cortex in mice. The yellow arrow indicates TUNEL/GAD67 double-positive cells. **k** Quantification of the number of TUNEL/GAD67 double-positive cells in the cerebral cortex in mice. The data are presented as the means ± SDs (*n* = 4 mice per group). Each dot in (**c**–**e,** **g,** **i**, **k**) denotes one mouse. Statistics calculated by unpaired two-tailed Student’s *t* test. Source data are provided as a Source Data file.

Based on our zebrafish findings, we decided to investigate sulindac’s effects on behavioural regulation and neuronal apoptosis in mammals. Therefore, the open field test was used to evaluate the locomotor activity of mice after one month of intragastric administration of sulindac (Fig. 6a). Strikingly, we observed that the mice exhibited hyperactivity and increased excitability, as estimated by their running distance, average velocity, and frequency of centre entry (Fig. 6b–e). Interestingly, this abnormal hyperactivity became more obvious with increasing doses of sulindac (26 mg/kg, 52 mg/kg and 104 mg/kg) in mice, which was similar to the phenomenon observed in zebrafish. Therefore, these results suggest that long-term use of sulindac can lead to hyperactivity in vertebrates.

To further confirm that the autophagic cell death of GABAergic neurons was also induced by sulindac in mice, haematoxylin/eosin (H&E) staining and Nissl staining were performed and revealed that sulindac administration for 1 month also resulted in significant neuronal cell death in mice at doses of 26 mg/kg, 52 mg/kg (human equivalent dose) and 104 mg/kg (Fig. 6f–i). Moreover, consistent with the observations in zebrafish, TUNEL assays revealed that the apoptosis of GAD67-positive cells increased significantly in mice (Fig. 6j, k). All these data imply that sulindac has a potentially toxic effect on GABAergic neurons, not only in lower vertebrates but also in mammals.

## Discussion

NSAIDs play an important role in clinical medicine. They are mostly used for the treatment of patients suffering from pain and inflammatory conditions such as chronic pain, osteoarthritis, rheumatoid arthritis, postoperative surgical conditions and menstrual cramps but are even used extensively as analgesics and antipyretics^49^. NSAIDs are also among the most frequently used drugs in the paediatric population^50,51^. However, after clinical application, an increasing number of NSAIDs have been reported to cause adverse effects, including damage to the cardiac^52^, gastrointestinal^53^, cardiovascular^54^ and neurological systems of patients^55,56^. Studies have also confirmed that overdose exposure to NSAIDs produces more serious risks to developing foetuses and children, who are even more sensitive than adults^57,58^. However, most research on the safety of NSAIDs has focused on foetal malformations and nephrotoxicity in children^13,59^. In contrast, there are few studies of neurodevelopmental disorders or brain damage caused by NSAIDs, and the mechanisms underlying their effects are still unclear. Here, we report that sulindac treatment induces autophagic cell death in GABAergic neurons via the PI3K/AKT/mTOR pathway, leading to hyperactive behaviour in zebrafish larvae and mice.

GABAergic neurons are a class of inhibitory neurons in the brains of vertebrates that are mainly distributed mainly in the mature midbrain region. They are involved in the regulation of multiple neural circuits, especially in regulating autonomic and motor behaviours^60^. Recently, it was reported that disrupting the normal development of GABAergic neurons during the early developmental stage of zebrafish leads to hyperactive motor behaviours^61,62^. Similarly, a study conducted on mice has demonstrated that dysfunction of ventral tegmental area GABA neurons can cause hyperactivity and manic-like behaviour^63^. In our study, we found that sulindac specifically induced apoptosis of GABAergic neurons in the midbrains of zebrafish larvae through the Akt pathway, resulting in enhanced locomotor behavioural activity in zebrafish larvae and mice. AKT, a serine/threonine kinase, has been reported to selectively induce developmental defects in GABAergic neurons after knockdown of Akt in mice^21^. In addition, inhibition of Akt pathway activity has been found to result in significantly impaired development of GABAergic neurons in zebrafish larvae^20^, which is in perfect agreement with our results. However, we were surprised to find that this restriction was reversed by overexpression of *akt1* mRNA or incubation with an AKT activator. Therefore, Akt is important for the development of GABAergic neurons, as it serves as a key regulator in the PI3K/Akt/mTOR pathway.

Sulindac, as a nonselective anti-inflammatory drug affecting COXs, is widely used for the treatment of osteoarthritis and rheumatoid arthritis. In recent years, an increasing number of studies have confirmed that there may be some COX-independent pathways involved in the pharmacological mechanism of sulindac^64,65^. In our study, we were surprised to find that sulindac selectively induced apoptosis of GABAergic neurons. However, the downstream mechanism of sulindac remains unclear and needs to be further explored. Interestingly, RXRα, which is a unique nuclear receptor of the RXR subfamily, was predicted to be a target of sulindac by the SEA database, and accumulating evidence indicates that RXRα also has extranuclear functions^66–68^. For example, RXRα migrates from the nucleus to the cytoplasm in response to differentiation^69^, survival^70^, apoptosis^71^ and inflammation^72^. Subsequently, we further confirmed strong binding between sulindac and RXRα via molecular docking and the SA-biotin system. As expected, the promoting effects of sulindac on autophagy and apoptosis were reversed by the RXRα competitive ligand SR11237, confirming that autophagy and apoptosis are directly related to the binding of sulindac and RXRα. This result is similar to the previously reported finding that sulindac can bind to RXRα and induce apoptosis in colon cancer^73^. The PI3K/AKT/mTOR pathway plays a major role in the processes of proliferation, apoptosis, and differentiation and is also closely related to the developing nervous system^74^. Our results showed that the PI3K/AKT/mTOR pathway was suppressed by sulindac. Moreover, both additional *akt1* mRNA or activation of mTOR reversed the effects of sulindac, further supporting the hypothesis that sulindac acts by inhibiting the PI3K/AKT/mTOR pathway in the autophagic cell death process of zebrafish neurons.

Autophagy is well known as a necessary process in cell physiology and is involved in regulating both cell survival and death^75^. Many studies have confirmed that autophagy has a bidirectional effect on apoptosis^76^. In our study, we found that the apoptosis of GABAergic neurons induced by sulindac was also accompanied by autophagy, and 3-MA, an inhibitor of autophagy, not only significantly attenuated sulindac-induced autophagy in larval zebrafish neurons but also significantly reduced neuronal apoptosis. However, the apoptosis inhibitor Z-VAD-FMK alleviated only apoptosis, not autophagy. This result suggests that sulindac treatment may activate autophagy to promote neuronal apoptosis. This is consistent with our above data that sulindac can inhibit the activity of the PI3K/AKT/mTOR pathway, leading to a reduction in the phosphorylation of mTOR (p- mTOR), followed by the occurrence of autophagy. Indeed, recent studies have revealed that the activation of the PI3K/Akt/mTOR pathway can induce autophagy via dephosphorylation of mTOR in several cell types^77,78^. Therefore, it is possible that the occurrence of autophagy induced by sulindac is responsible for the apoptosis of GABAergic neurons.

**Fig. 7:**
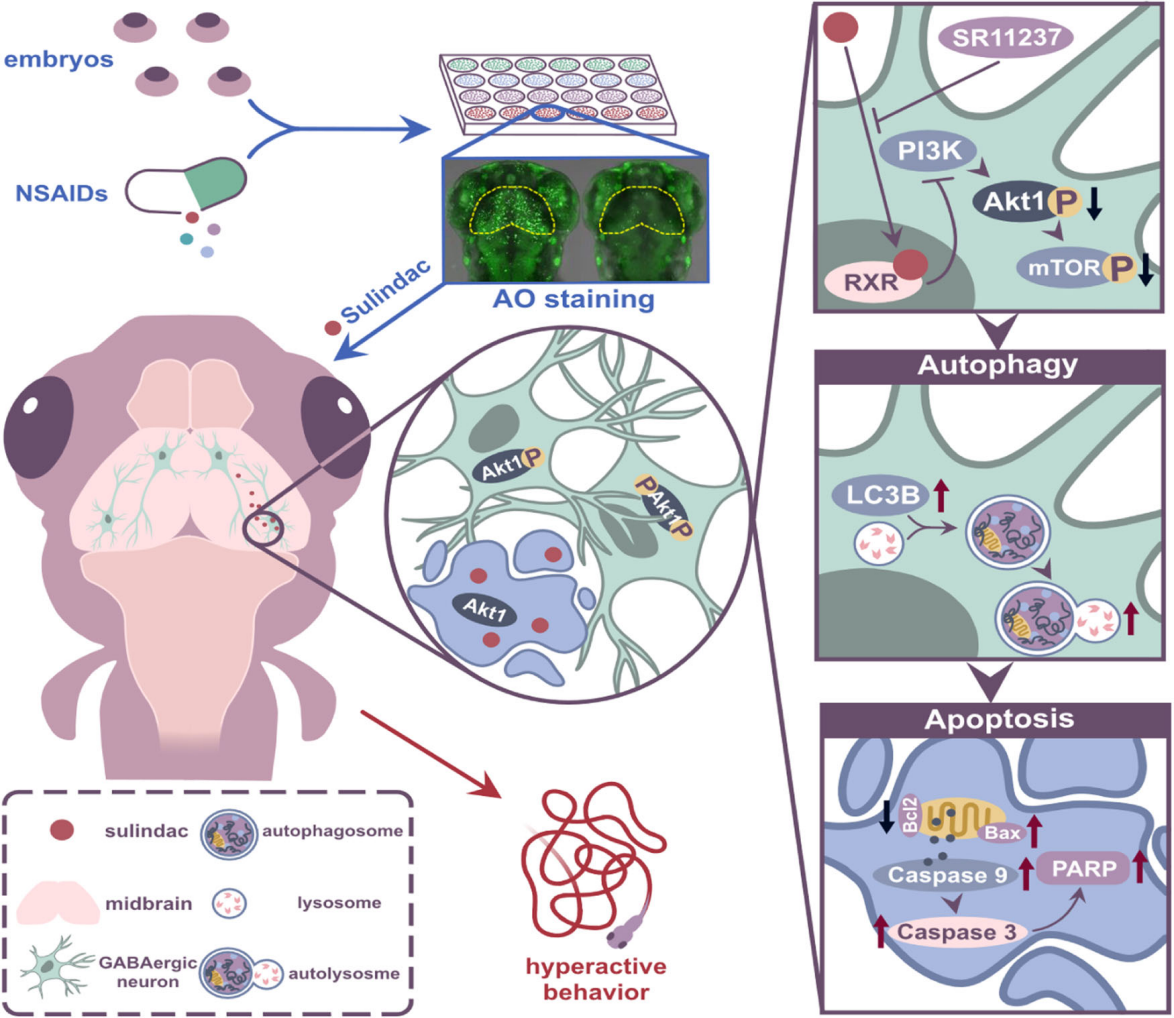
Schematic illustration of the proposed mechanisms. Sulindac treatment induces RXRa-dependent autophagic cell death in GABAergic neurons via the PI3K/AKT/mTOR pathway, leading to hyperactive behaviour in zebrafish larvae.

In conclusion, our study demonstrates that sulindac triggers autophagy-mediated apoptosis of GABAergic neurons via the PI3K/AKT/mTOR pathway, resulting in hyperactive behaviour in zebrafish larvae and mice (Fig. 7). These findings suggest that prolonged use of sulindac may lead to neurotoxicity during CNS development. Thus, these results deepen our understanding of the effects of sulindac, especially in the nervous system, and should encourage more rational and effective treatments to be employed in the clinic.

## Methods

### Ethical statement

All experimental procedures were approved by the Institutional Animal Care and Use Committee of the South China University of Technology (approval number SCUT-2019-079).

### Zebrafish maintenance

The zebrafish strains were maintained in standard laboratory conditions. The Tuebingen strain of zebrafish was used to obtain wild-type embryos, which were maintained in E2 medium at 28.5 °C and staged as previously reported^79^. The following mutant and transgenic lines were used: *p53*^*M214K*^^80^, *Tg(Xla.Tubb:DsRedx)*^81^, *Tg(Xla.Tubb:bcl-2)*^82^, *Tg(gad1b:mCherry)*^83^, *Tg(vglut2a:GFP)*^83^, *Tg(atoh7: mCherry)*^84^ and *Tg(islet:GFP)*^85^. All experiments involving zebrafish were approved by the Institutional Animal Care and Use Committee of the South China University of Technology.

### Drug exposure

Zebrafish embryos at 2 dpf were soaked in egg water containing chemicals for 2 days, and relevant experiments were performed at 4 dpf. DMSO (Sigma‒Aldrich #D8418), sulindac (Selleck #S2007, 20 μM), SR11237 (Sigma‒Aldrich #S8951, 1 μM), CD3254 (MCE #HY-107399, 1 μM), 3-methyladenine (MCE, #HY-19312, 1 mM), Z-VAD-FMK (Selleck #S7023, 100 μM), dmPGE2 (Apexbio #B7568, 10 μM), MHY1485 (MCE #HY-B0795, 5 μM), SC97 (Selleck #S7863, 1 μM) and MK-2206 (Selleck #S1078, 30 μM) were used in this study.

### AO staining, TUNEL, H&E staining and Nissl staining

For AO staining, embryos recovered at 4 dpf were incubated in 10 μg/mL AO stain (Sigma‒Aldrich) dissolved in embryo medium in the dark for ~30 min. TUNEL staining was performed with an In Situ Cell Death Detection Kit TMR red (#12156792910, Roche) as previously described^86^. H&E staining was conducted to observe the morphological and quantitative changes in neurons. Nissl staining was used to determine the loss of Nissl substance and the number of Nissl bodies. Both H&E staining and Nissl staining were performed according to standard protocols.

### Immunostaining

Embryos were collected and fixed overnight at 4 °C with 4% paraformaldehyde, then briefly permeabilized with methanol. Subsequently, they were rinsed and washed in PBST (PBS containing 0.1% Tween-20) to eliminate residual methanol. After blocking with blocking buffer (0.5% Triton-X PBS, 5% lamb serum, and 1% DMSO) for 2 h at room temperature, embryos were incubated with primary antibody in blocking buffer for 2 days, followed by secondary antibody for another day at 4 °C. Rabbit anti-Akt1 (Invitrogen, #PA5-29169, 1:100) and mouse anti-mCherry (Abcam, #ab125096, 1:500) were used as primary antibodies, and Alexa Fluor 488-conjugated anti-rabbit (Invitrogen, #A32731, 1:400) and Alexa Fluor 555-conjugated anti-mouse (Invitrogen, #A32727, 1:400) were used as secondary antibodies.

### Single-cell gene expression quantification

The raw sequencing data were processed using Cell Ranger (v5.0) provided by 10X Genomics with the default options. The reads were aligned to the zebrafish reference transcriptome (Ensembl release 104). To ensure data quality, reads with low-quality barcodes and unique molecular identifiers (UMIs) were filtered out. For UMI counting, only reads that uniquely mapped to the transcriptome and intersected an exon by at least 50% were considered. Prior to quantification, UMI sequences were corrected for sequencing errors, and valid barcodes were identified using the EmptyDrops method^87^. The resulting cell-by-gene matrices were generated through UMI counting and cell barcode assignment.

### Dimensionality reduction and clustering

The resulting matrix was imported into Seurat^88^ (v3.1.1) for downstream analysis. Briefly, cells that met the filter parameters of gene numbers between 200 and 3000, UMI counts <20000, and mitochondrial counts <15% were retained for subsequent analysis. Additionally, doublet GEMs were filtered out using DoubletFinder^89^ (v2.0.3) with a default estimated doublet rate of 7.5%. After retaining a total of 7,758 high-quality cells, the expression values were normalized using a global-scaling method known as “Log Normalize”, which involved multiplying the total expression by a scale factor (default 10,000) and subsequently log-transforming the results. To account for batch effects and behavioural conditions on clustering, we employed Harmony to aggregate all samples^90^. The Harmony algorithm takes as input a principal component analysis (PCA) embedding of cells and their corresponding batch assignments and returns a batch-corrected embedding. We performed a random permutation of a subset of the data (default: 1%), reran PCA to create a null distribution of gene scores and repeated this procedure. The identification of PCs was based on their enrichment of genes with low p values, indicating their relevance for downstream clustering and dimensional reduction^91^. Euclidean distances between cells were calculated based on previously identified PCs. In brief, Seurat uses a shared-nearest neighbour (SNN) graph to embed cells, where edges are drawn between cells with similar gene expression patterns. To partition this graph into highly interconnected quasi-cliques or communities, we constructed the SNN graph based on the Euclidean distance in PCA space and refined the edge weights between cells based on the shared overlap in their local neighbourhoods (Jaccard distance). The Louvain^92^ algorithm was employed for cell clustering and subclustering, while nonlinear dimensionality reduction (t-SNE) was used for data visualization. Subsequently, cell annotation was performed using semisupervised methods such as singleR and manual annotation. We identified and clustered four major cell types, including optic neurons, NSPCs, postmitotic neurons, and nonneuronal cells. Furthermore, the neuronal population within the brain was reclustered into seven distinct subpopulations, resulting in a total of 22 clusters. Supplementary Table 3 provides detailed information about the cells within each cluster.

### Differential expression analysis

The Wilcoxon rank-sum test^93^ (Seurat version 3.1.1) was used for the differentially expressed gene analysis in this study. The expression values of each gene within a given cluster were compared with the rest of the cells. Significant genes were identified based on three main criteria: the genes were at least 1.28-fold overexpressed in the target cluster, the genes were expressed in more than 25% of the cells belonging to the target cluster, and the p value was less than 0.05.

### Morpholino oligonucleotide (MO) injection

A previously described morpholino oligonucleotides (MO) to ptgs1/cox-1 (5ʹ-TCAGCAAAAAGTTACACTCTCTCAT-3ʹ)^41^, ptgs2a/cox-2a (5ʹ-AACCAGTTTATTCATTCCAGAAGTG-3ʹ)^41^ and ptgs2b/cox-2b (5ʹ-AGGCTTACCTCCTGTGCAAACCACG-3ʹ)^94^ were purchased from Gene Tools. The MOs for ptgs1, ptgs2a, and ptgs2b were injected into zebrafish embryos at the one-cell stage at 300 μM.

### Synthesis of mRNA and guide RNA

The Akt coding sequence was amplified using primers (Akt1, 5ʹ-ATGGCGACAGATGTG-3ʹ and 5ʹ-TCATGCTGTTCCGCT-3ʹ; Akt2, 5ʹ-ATGAACGAGATCAGCGTCGTCA-3ʹ and 5ʹ-TCACTCCCGCACACTGGC-3ʹ; Akt3a, 5ʹ-ATGAGCGATGTCACCGTCG-3ʹ and 5ʹ-TCATTCTCTCCCGCTGGCCGA-3ʹ; Akt3b, 5ʹ-ATGAACGACCTGAACGTCGTG-3ʹ and 5ʹ-TCACTCCCGCCCGCTG-3ʹ), and the PCR product was inserted into the pCS2+ vector. After linearization by Not1, the capped Akt (Akt1, Akt2, Akt3a and Akt3b) mRNA was synthesized in vitro by using an mMESSAGE mMACHINE® SP6 Kit (Invitrogen, #01112989) according to the manufacturer’s instructions. The mRNAs were injected into each embryo at a concentration of 200 pg for Akt1, 150 pg for Akt2, and 100 pg each for Akt3a and Akt3b.

The gRNA target sites were designed using online tools (http://chopchop.cbu.uib.no/). The primers used for amplification of gRNA templates were as follows: T7-akt1-gRNA-F: 5ʹ-TAATACGACTCACTATAGGGACAATGATTACGGTCGTGGTTTTAGAGCTAGAAATAGC-3ʹ; gRNA-R: 5ʹ-AGCACCGACTCGGTGCCACT-3ʹ. gRNA was transcribed using a MAXIscript T7 kit (Invitrogen, #AM1314). The Cas9 protein (NEB, Cat# M0646T), an RNA-guided endonuclease, was used to cleave the site-specific genomic DNA^95^. A volume of 1 nL of a mixture of gRNA (100 pg) and Cas9 protein (500 pg) was coinjected into each single-celled embryo.

### Plasmid construction

For the construction of the cmv:RFP-Lc3 plasmid, PCR was performed to generate RFP cDNA without the termination codon, and then replace the EGFP coding region of pEGFP-C1, and the resulting plasmid was named RFP-C1. The fragment of Lc3 was inserted into the corresponding sites in the RFP-C1 plasmid. Finally, a 30-pg plasmid was injected into one-cell-stage embryos. For the construction of the tissue-specific CRISPR vector, we sought assistance from Professor Yang’s laboratory^32^. The *gad1b* promoter from the transgenic line *Tg(gad1b:mCherry)* was cloned and inserted into *Cas9-T2A-mCherry,U6:gRNA(null)* to create the *gad1b:Cas9-T2A-mCherry,U6:gRNA(null)* vector by Gibson assembly reaction^96^. The sequence of the *akt1* gRNA target was then inserted into the U6:gRNA cassette to construct the CRISPR vector *gad1b:Cas9-T2A-mCherry,U6:gRNA akt1*. Finally, a mixture of 100 pg of CRISPR vector and 20 pg of Tol2 mRNA was injected into one-cell-stage wild-type embryos.

### Confocal imaging

Zebrafish larvae were anaesthetised with 0.01% tricaine and mounted in 1% low-melting agarose with 0.01% tricaine. A Zeiss 800 confocal microscope was used for imaging the samples. Optical sections were obtained under a ×10 or ×20 air immersion objective lens at a resolution of 1024 × 1024 pixels, with the pinhole diameter adjusted to 1 Airy unit for each emission channel to ensure all intensity values fell within the linear range of 1 to 250. A 561-nm laser with an emission wavelength range of 570–650 nm and a 488-nm laser with an emission wavelength range of 500–550 nm were utilized. The microscope was operated on the Zeiss Zen blue 2.5 software platform (Carl Zeiss).

### Detection of mitochondrial membrane potential

After drug treatment, whole-brain tissue was separated from zebrafish juveniles, and a cell suspension was prepared according to a previously described method^97^. Briefly, brain tissues were dissociated with 300 μL papain solution (28 units/mL, Worthington) containing 1% DNase and 12 mg/mL L-cysteine in DMEM/F12. The mixture was incubated at 37 °C for 15 min with gentle agitation. Dissociated cells were then washed twice with washing buffer (100 mL: 650 μL 45% glucose, 500 μL 1 M HEPES and 5 mL FBS into 93.85 mL 1×DPBS). Finally, the collected cells were incubated with JC-1 (10 mM) in the dark at 37 °C for 30 min. Finally, the changes in mitochondrial membrane potential (ΔΨm) were detected using flow cytometry (Beckman Coulter, USA).

### Extraction of cytoplasm and mitochondria from cells

First, the cell suspension was prepared according to a previously described method^97^. Briefly, brain tissues were dissociated with 300 μL papain solution (28 units/mL, Worthington) containing 1% DNase and 12 mg/mL L-cysteine in DMEM/F12. The mixture was incubated at 37 °C for 15 min with gentle agitation. Dissociated cells were then washed twice with washing buffer (100 mL: 650 μL 45% glucose, 500 μL 1 M HEPES and 5 mL FBS into 93.85 mL 1×DPBS). Subsequently, a mitochondria isolation kit for tissue (Abcam, #ab110168) was used to obtain the cytoplasmic and mitochondrial fractions according to the manufacturer’s instructions with minor changes. Neuronal cells were homogenized in ice-cold mitochondria isolation buffer containing protease inhibitor cocktail after resuspension and then centrifuged at 1000 × *g* for 10 min twice at 4 °C to remove debris. The supernatant was centrifuged at 12,000 × *g* for 15 min twice to isolate the mitochondrial fraction. Finally, the resulting pellet containing mitochondria was washed twice with isolation buffer, and the remaining supernatant served as the cytosolic fraction.

### Western blotting

The encephalons of 4-dpf larvae were dissected and lysed in RIPA lysis buffer supplemented with protease inhibitor cocktail (Roche, #04693116001) for Western blot analysis. The proteins were then separated by 10–13.5% SDS‒PAGE and transferred to nitrocellulose (NC) membranes. The membranes were first blocked in 5% BSA and then incubated overnight at 4 °C with primary antibodies. Subsequently, the membranes were incubated with secondary antibodies for another 3 h. Images were visualized with a MiniChemi image system (Beijing Sage Creation Science Co., China). All the information on primary antibodies is provided in Supplementary Table 4.

### Detection of the interaction between sulindac and RXRα protein

A streptavidin–biotin (SA–biotin) system was utilized to confirm the interactions between sulindac and RXRα. Sulindac was biotinylated via a chemical reaction between the amino group of biotin and the carboxyl group of sulindac (Supplementary Fig. 14). Then, zebrafish embryos at 2 dpf were soaked in egg water containing biotinylated sulindac (20 μM) for 2 days. The encephalons of 4-dpf larvae were dissected and lysed in RIPA lysis buffer supplemented with protease inhibitor cocktail (Roche, #04693116001). The protein supernatant was obtained through low-temperature centrifugation. Subsequently, the protein mixture was transferred into a 1.5 mL microcentrifuge tube containing extensively washed streptavidin magnetic beads (Thermo Fisher, #88816) and incubated for 1.5 h at room temperature. Next, a magnetic stand was utilized to acquire target protein according to the instructions of the magnetic beads. Western blotting was used to analyse the target protein.

### Determination of locomotor behaviour in larvae

All locomotor behaviour detection was performed in 24-well plates during the light phase between 9 a.m. and 4 p.m. After exposure to sulindac (5 μM, 10 μM, 20 μM) or DMSO (0.1%) at 5 dpf, the free-swimming activities of zebrafish larvae in response to a 10-min light-to-dark photoperiod stimulation were recorded with a DanioVision Observation Chamber System (Noldus IT, Netherlands). The videos were analysed using EthoVision XT software, and the total distances moved together with mean velocity were subjected to statistical analysis.

### Mouse experiments

Four-week-old KM mice were obtained from Guangdong Medical Laboratory Animal Centre, Guangzhou, China. The animal licence number was SCXK (YUE) 2022-0002. All mice were housed in a facility with a light cycle running from 06:00 to 18:00, and the temperature was maintained at 20–22 °C with humidity at 50%. Food and water were available ad libitum. The mice were randomly divided into four groups (*n* = 10 per group, 5 male/5 female): saline group; sulindac groups with doses of 26 mg/kg, 52 mg/kg (human equivalent dose), and 104 mg/kg. All the mice received either sulindac or saline through gastric feeding. The treatment was repeated daily for the specified duration. After 30 days of gavage administration, behavioural monitoring experiments were performed. Subsequently, the mice were euthanized by administering with carbon dioxide for 2 min from a compressed gas tank followed by cervical dislocation, and the entire brain tissue of the mice was collected and fixed with a 4% paraformaldehyde solution for further experiments. To prepare paraffin sections, the fixed brain tissue was dehydrated with 70, 80, 95 and 100% alcohol followed by transparent with xylene, embedded in paraffin and cut into 5 μm thickness with a microtome (Leica, Germany). For immunostaining analysis of apoptotic GAD67-positive cells, the In Situ Cell Death Detection Kit, Fluorescein (Roche, #11684795910) was used along with a polyclonal rabbit antibody to GAD67 (Abcam, #ab97739, 1:100) and anti-rabbit IgG Alexa Fluor™ 555 conjugated antibody (Invitrogen, # A31572, 1:400). The sections were mounted with VECTASHIELD containing DAPI (Vector Laboratories, #H1200). These samples were observed under an FV-1000D microscope (Olympus), and the numbers of TUNEL/GAD67 double-positive cells were counted. All animal experiments in this study were performed in strict accordance with the institutionally approved protocol according to the USPHS Guide for the Care and Use of Laboratory Animals and with the approval of the Institutional Animal Care and Use Committee of the South China University of Technology.

### Locomotor activity

After one month of intragastric gavage, the locomotor activity of mice was evaluated in an open field according to a previously reported method^98^. Briefly, each mouse was placed in a plastic chamber divided into a central arena and a peripheral arena with dimensions of 50 cm × 50 cm × 50 cm, length × width × height and allowed to freely explore during a 5-min session. All tests were performed in a sound-attenuated room and mouse movements were tracked with a video-tacking camera positioned above the arena using behavioural analysis software (Tracking Master V4.10 Software, Zhongshi Co., Ltd).

### Statistics and reproducibility

The data are presented as the means ± standard deviations (SDs), with sample sizes (n) provided in the text or figure legend for each experiment. Each experiment was independently repeated at least three times. All statistical analyses were performed using GraphPad Prism version 8. Two-tailed unpaired Student’s *t*-tests were employed for comparisons between two groups, unless otherwise specified. The densities of the western blot bands were analyzed by ImageJ software.

### Reporting summary

Further information on research design is available in the Nature Portfolio Reporting Summary linked to this article.

### Supplementary information

Supplementary information

Reporting Summary

### Source data

Source data

## Data Availability

All relevant data supporting this study are available within the paper and its supplementary information. The raw scRNA-Seq data had been deposited to the GEO database by the accession number GSE212888. Ensembl release 104 [https://ftp.ensembl.org/pub/release-104/gtf/danio_rerio/] was used to define genes for zebrafish. Source data are provided with this paper.
