# Supplementary material for: Sulindac selectively induces autophagic apoptosis of GABAergic neurons and alters motor behaviour in zebrafish
Source: Nat Commun. 2023 Sep 2;14:5351. doi: 10.1038/s41467-023-41114-y (PMC10475106; doi:10.1038/s41467-023-41114-y)
Supplement: Supplementary file 1 — Supplementary information [file 41467_2023_41114_MOESM1_ESM.pdf]

Supplementary information

Sulindac selectively induces autophagic apoptosis of GABAergic neurons and alters motor behavior in zebrafish

Wenwei Sun[1,7], Meimei Wang[1,7], Jun Zhao[1], Shuang Zhao[1], Wenchao Zhu[2], Xiaoting Wu[3], Feifei Li[1], Wei Liu[1], Zhuo Wang[1], Meng Gao[2], Yiyue Zhang[1], Jin Xu[1], Meijia Zhang[1], Qiang Wang[1], Zilong Wen[4,5], Juan Shen[3*,] Wenqing Zhang[1,6*,] Zhibin Huang[1*]

[1]Division of Cell, Developmental and Integrative Biology, School of Medicine, South China University of Technology, Guangzhou 510006, China.

[2]National Engineering Research Center for Tissue Restoration and Reconstruction, Key Laboratory of Biomedical Engineering of Guangdong Province, Key Laboratory of Biomedical Materials and Engineering of the Ministry of Education, Innovation Center for Tissue Restoration Reconstruction, South China University of Technology, Guangzhou 510006, China.

[3]Guangdong Provincial Key Laboratory of Pharmaceutical Bioactive Substances, Guangdong Pharmaceutical University, Guangzhou 510006, China.

[4]Division of Life Science, State Key Laboratory of Molecular Neuroscience and Center of Systems Biology and Human Health, the Hong Kong University of Science and Technology, Clear Water Bay, Kowloon, Hong Kong, People's Republic of China.

[5]Greater Bay Biomedical Innocenter, Shenzhen Bay Laboratory, Shenzhen Peking University-Hong Kong University of Science and Technology Medical Center, Shenzhen 518055, China.

[6]Greater Bay Biomedical Innocenter, Shenzhen Bay Laboratory, Shenzhen 518055, China.

[7]These authors contributed equally: Wenwei Sun, Meimei Wang.

[*]**Correspondence:** huangzhb1986@scut.edu.cn (Z. B. H.), mczhangwq@scut.edu.cn (W. Q. Z.), shenjuan0412@126.com (J. S.).

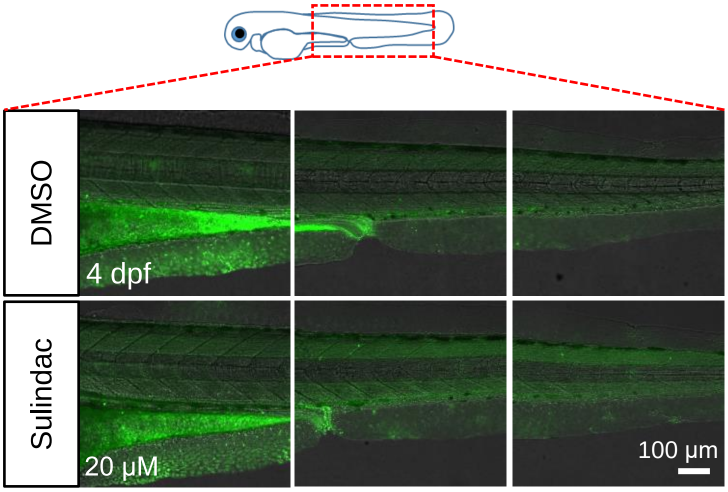

Supplementary Fig. 1 (related to Fig. 1) Sulindac did not cause apoptosis in the trunk region of zebrafish after treatment with DMSO or sulindac. AO staining revealed an indifference of apoptosis in the trunk region of zebrafish after treatment with DMSO or sulindac. The experiment was repeated twice with similar results, and five zebrafish were observed in each group.

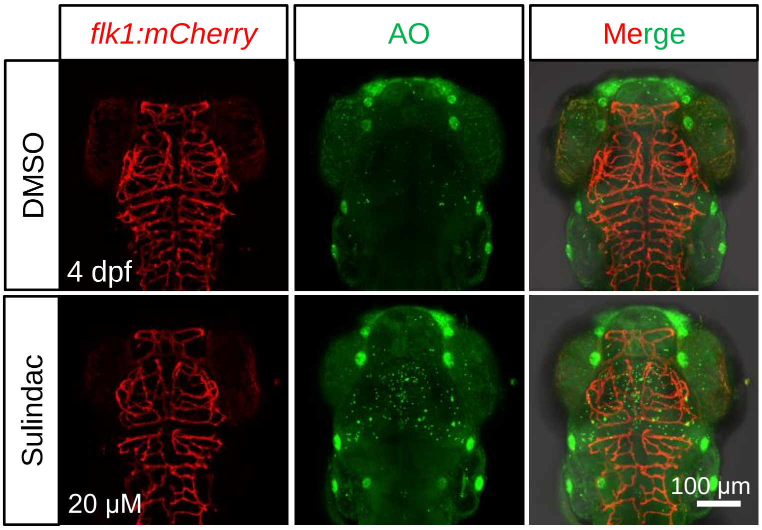

Supplementary Fig. 2 (related to Fig. 1) The apoptosis cells were not cerebrovascular cells after treatment with DMSO or sulindac in zebrafish larvae. AO staining revealed that the apoptosis cells (green signals) did not colocalize with cerebrovascular cells (red signals) in the midbrains of *Tg(flk1:mCherry)* embryos after treatment with DMSO or sulindac. The experiment was repeated once with similar results, and five zebrafish were observed in each group.

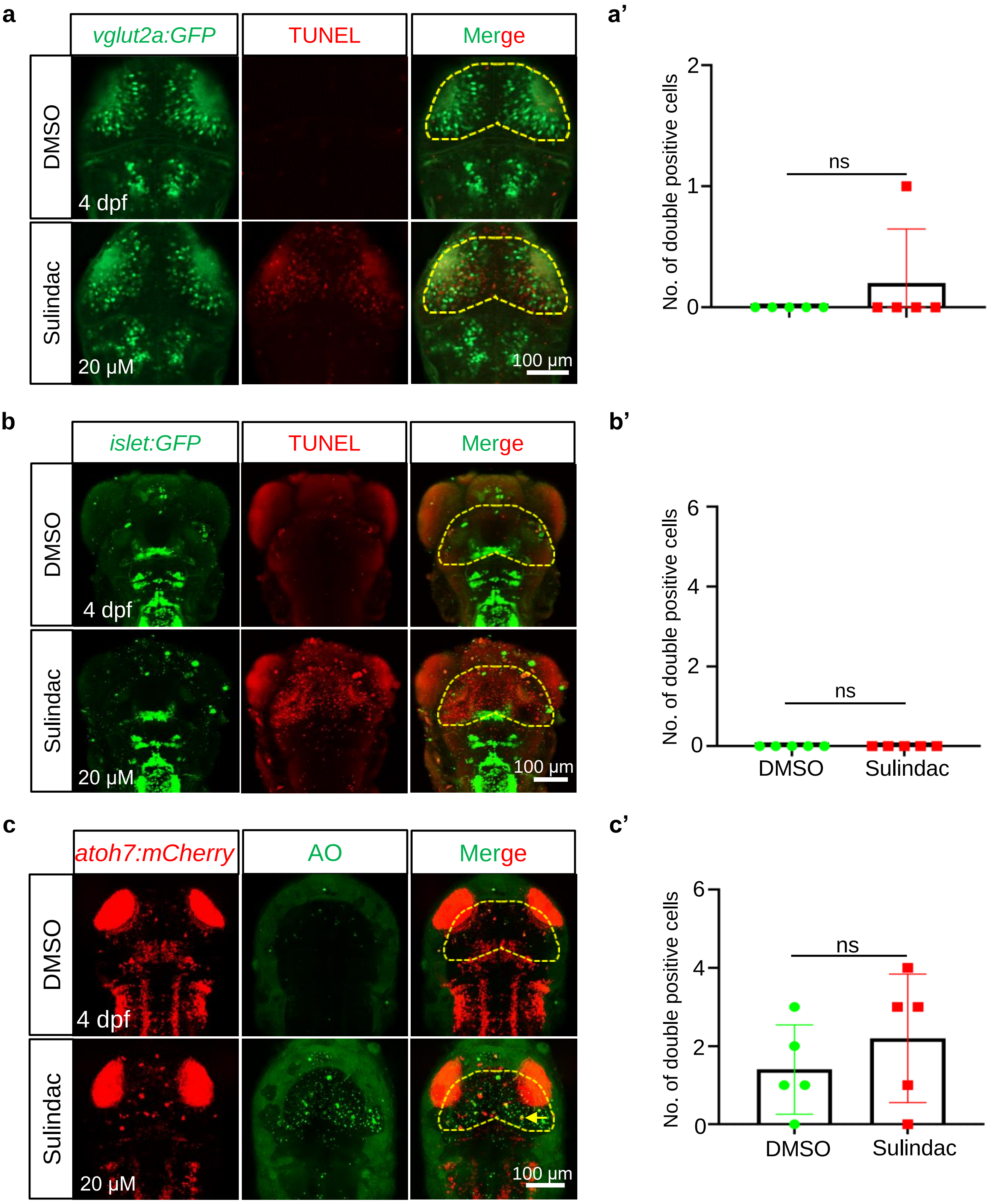

Supplementary Fig. 3 (related to Fig. 2) Sulindac-induced apoptotic neurons were not glutamatergic neurons, retinal ganglion cells or cranial motor neurons. (**a**) TUNEL staining showed that the apoptosis neurons did not colocalize with vglut2a:GFP^{+} glutamatergic neurons in the midbrain after treatment with DMSO or sulindac. (**a'**) Quantification of the double-positive cells indicated by dashed lines in the midbrains of embryos. (**b**) TUNEL staining showed that the apoptosis neurons did not colocalize with islet:GFP^{+} cranial motor neurons in the midbrain after treatment with DMSO or sulindac. (**b'**) Quantification of the double-positive cells indicated by dashed lines in the midbrain of embryos. (**c**) AO staining revealed that the apoptosis neurons did not colocalize with atoh7:mCherry^{+} atoh7+ neurons in the midbrain of embryos after treatment with DMSO or sulindac. (**c'**) Quantification of the double-positive cells indicated by dashed lines in the midbrains of embryos. All the data are presented as the means ± SDs (*n* = 5 larvae for per group). Each dot in (**a'**, **b'** and **c'**) denotes one larva. NS, no significance. Statistics calculated by unpaired two-tailed Student's *t*-test.

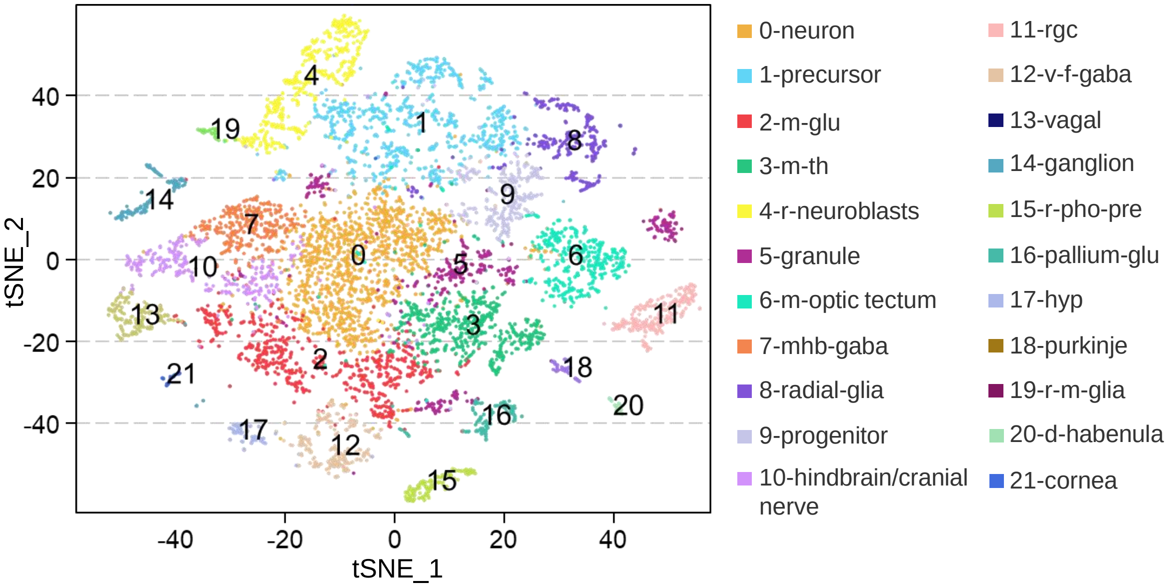

Supplementary Fig. 4 (related to Fig. 3) The t-SNE plot of the pooled cells was aggregated into 22 clusters, marked by numbers with different colors, and cluster annotations were shown in table S2.

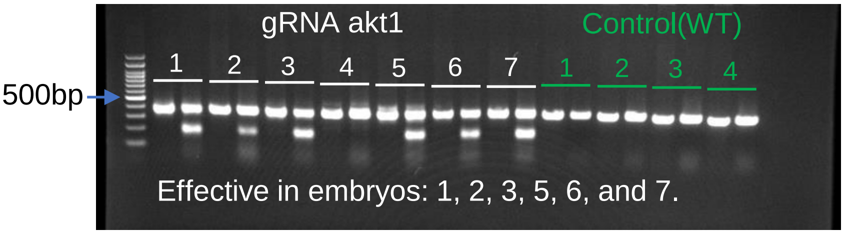

Supplementary Fig. 5 (related to Fig. 3d and e) Identification of efficient CRISPR target sequences for *akt1*. Mix and inject the gRNA *akt1* with Cas9 protein into one-cell stage WT strain embryos. Extract DNA from injected embryos at 24 hpf by lysis buffer and test the efficiency of *akt1* target sequences by T7E1 enzyme digestion.

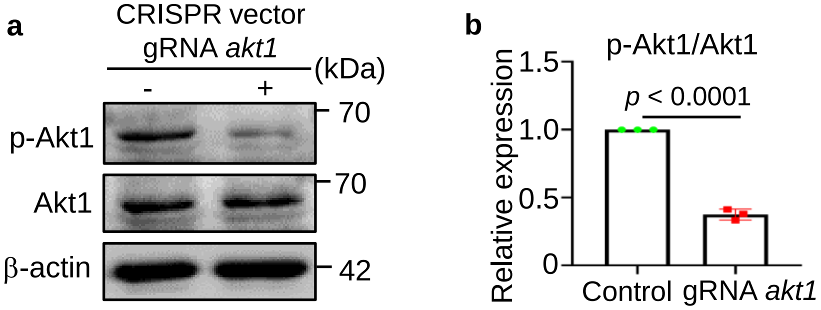

Supplementary Fig. 6 (related to Fig. 3d and e) Detection of knockout efficiency of CRISPR vector. (**a**) After injection of the tissue-specific CRISPR vector, Western blot analysis showed that the protein level of p-Akt1 in the brain tissue was significantly decreased at 4 dpf. (**b**) The data are presented as the means ± SDs (n = 3 independent biological replicates). Statistics calculated by unpaired two-tailed Student's t test. Source data are provided as a Source Data file. β-actin was used as an internal control.

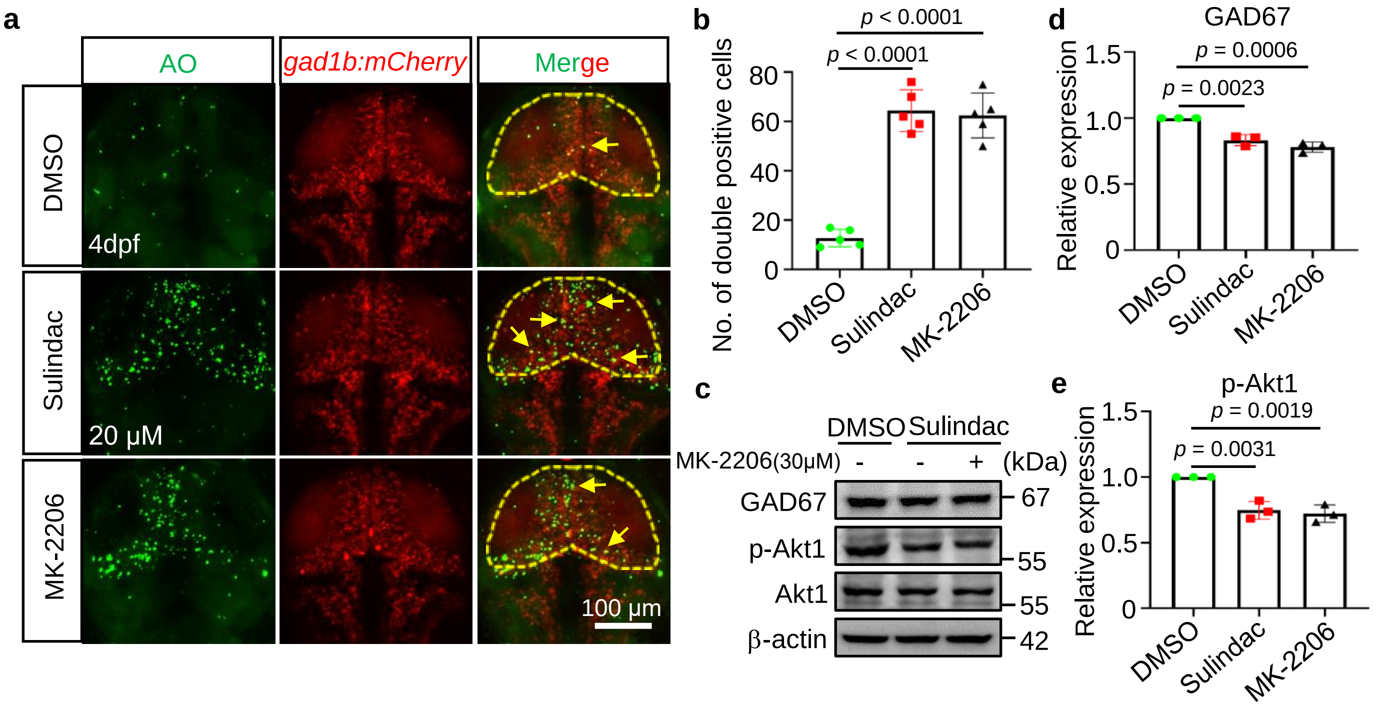

Supplementary Fig. 7 (related to Fig. 3) Akt1 inhibitor could induce the apoptosis of GABAergic neurons. (**a**) The number of apoptotic GABAergic neurons was increased obviously after Akt1 inhibitor mk-2206 treatment. (**b**) Quantification of the double-positive cells indicated by dashed lines in the midbrains of embryos. Each column represents the mean ± SDs (n = 5 larvae for per group, each dot denotes one larva). Statistics calculated by unpaired two-tailed Student's *t* test. Source data are provided as a Source Data file. **(c**) Western blot analysis showed the protein level of GAD67 and p-Akt1 were significantly decreased after sulindac or MK-2206 (30 μM) treatment. (**d** and **e**) The data are presented as the means ± SDs (n = 3 independent biological replicates). Statistics calculated by unpaired two-tailed Student's *t* test. Source data are provided as a Source Data file. β-actin was used as an internal control.

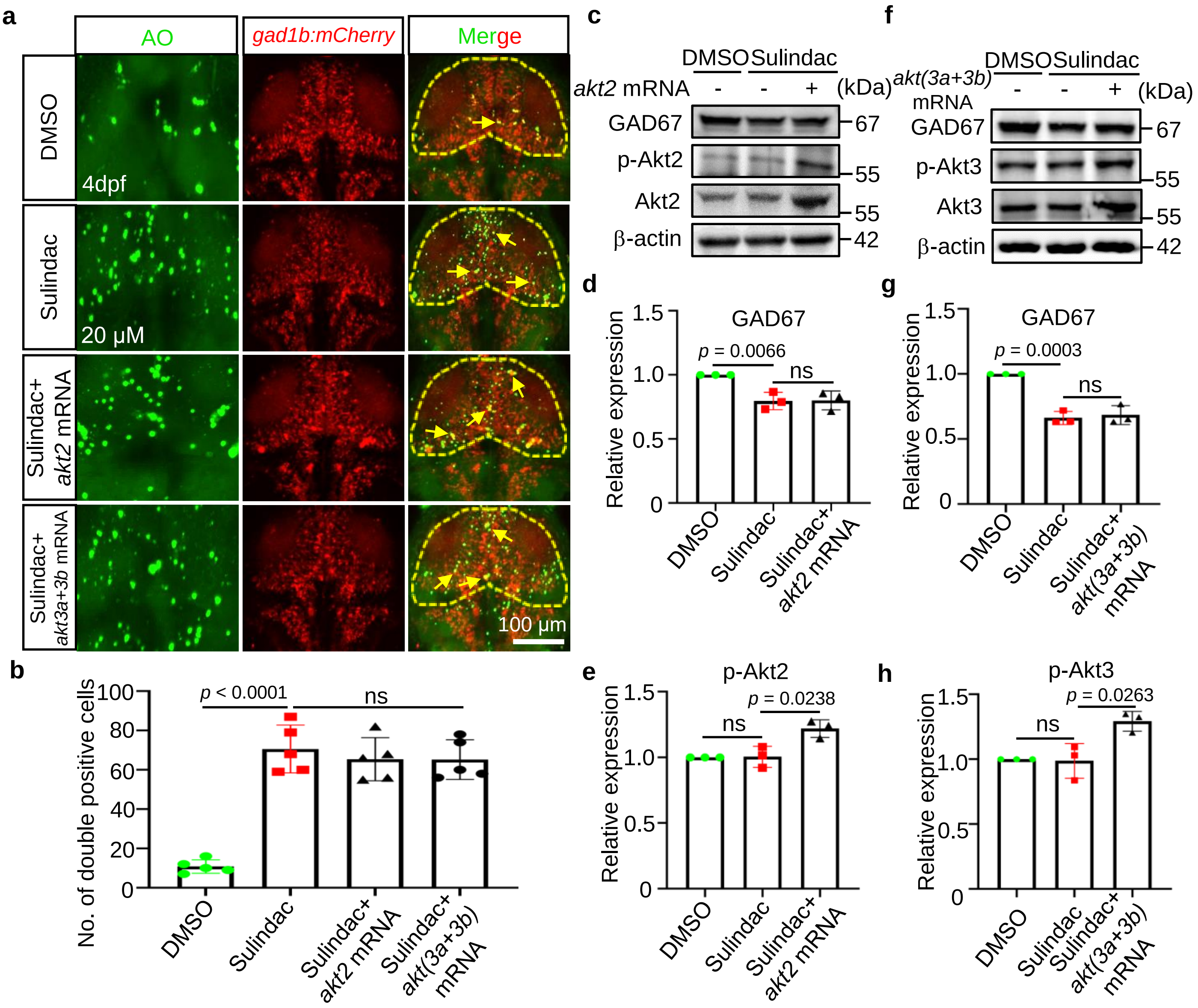

Supplementary Fig. 8 (related to Fig. 3) Overexpression of *akt2* mRNA or *akt3* mRNA failed to rescue sulindac-induced apoptotic GABAergic neurons. (**a**) The number of apoptotic GABAergic neurons was not rescued by *akt2* mRNA or *akt(3a+3b)* mRNA. (**b**) Quantification of the double-positive cells indicated by dashed lines in the midbrains of embryos. Each column represents the mean ± SDs (*n* = 5 larvae for per group, each dot denotes one larva). Statistics calculated by unpaired two-tailed Student's *t* test. Source data are provided as a Source Data file. (**c** and **f**) Western blot assays confirmed that overexpression of *akt2* mRNA or *akt(3a+3b) mRNA* could not reverse the decrease of GAD67 protein induced by sulindac. (**d**, **e**, **g** and **h**) The data are presented as the means ± SDs (*n* = 3 independent biological replicates). Statistics calculated by unpaired two-tailed Student's *t* test. Source data are provided as a Source Data file. β-actin was used as an internal control.

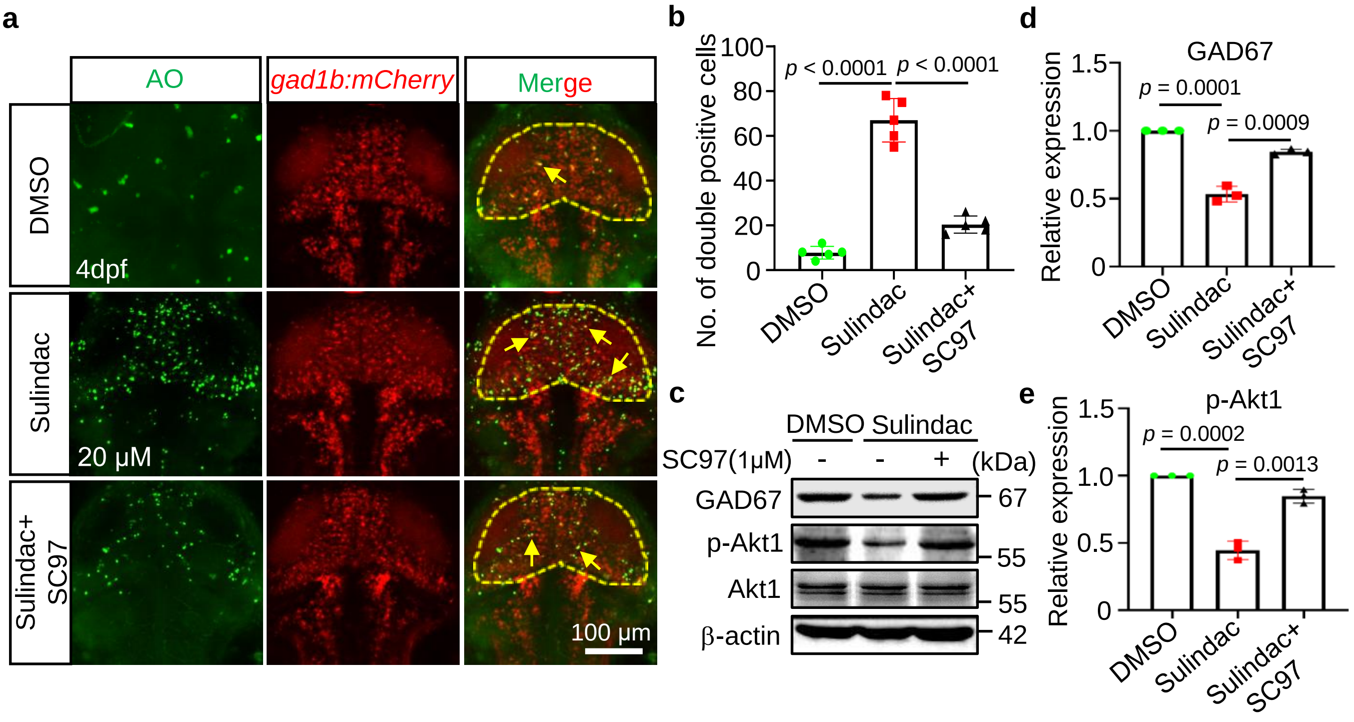

Supplementary Fig. 9 (related to Fig. 3) Sulindac-induced GABAergic neuronal apoptosis could be restored by SC97. (**a**) The number of apoptotic GABAergic neurons was rescued by Akt1 agonist SC97 (1μM). (**b**) Quantification of the double-positive cells indicated by dashed lines in the midbrains of embryos. Each column represents the mean ± SDs (n = 5 larvae for per group, each dot denotes one larva). Statistics calculated by unpaired two-tailed Student's t test. Source data are provided as a Source Data file. (**c**) Western blot analysis showed that the decrease of GAD67 and p-Akt1 protein induced by sulindac was significantly rescued by SC97 at 4 dpf. (**d** and **e**) The data are presented as the means ± SDs (n = 3 independent biological replicates). Statistics calculated by unpaired two-tailed Student's t test. Source data are provided as a Source Data file. β-actin was used as an internal control.

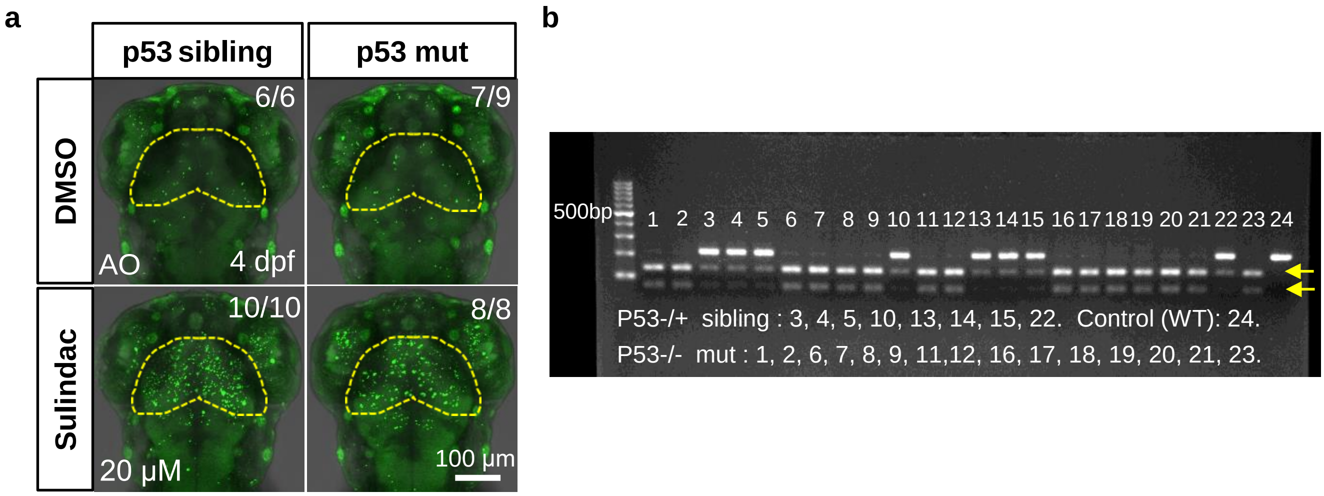

Supplementary Fig. 10 (related to Fig. 4) Sulindac induced apoptosis of zebrafish neurons by a p53-independent pathway. (**a**) AO staining revealed that knockout p53 did not rescue the neuronal apoptosis in the midbrain of zebrafish embryos after treatment with sulindac. The midbrain is indicated by dashed lines. (**b**) The p53 mutant embryos were identified by PCR amplification of the mutation site and digested with MboII enzyme into two short fragments (yellow arrow).

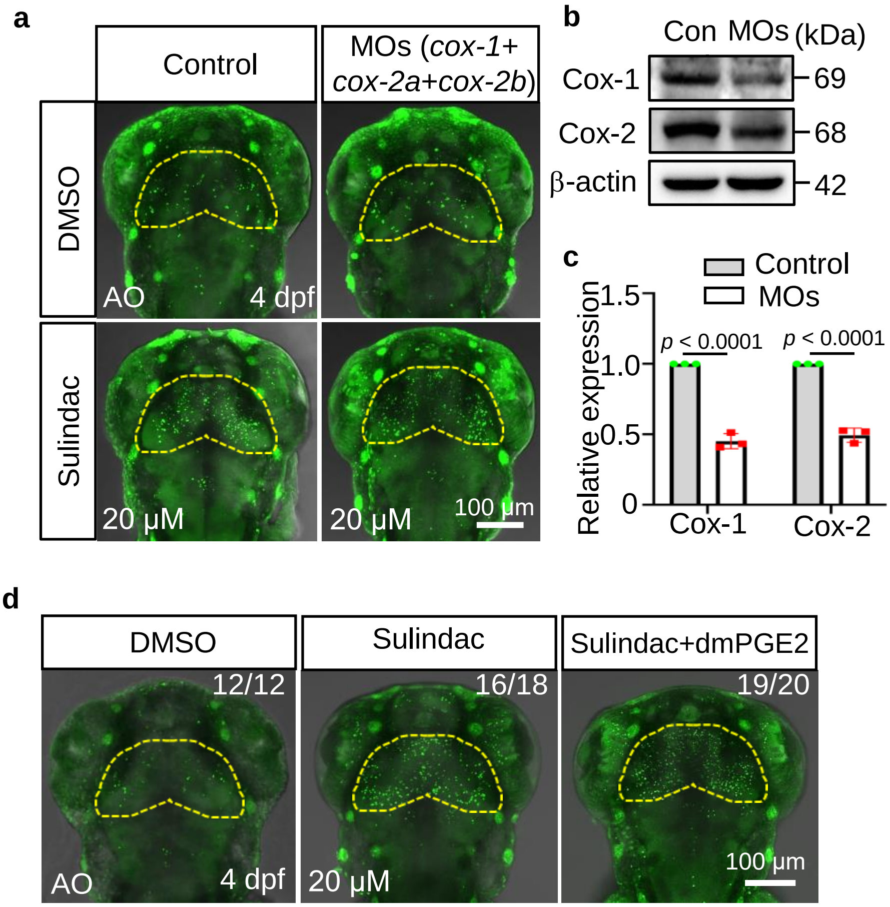

Supplementary Fig. 11 (related to Fig. 4) Sulindac induced apoptosis of zebrafish neurons by a COXs-independent pathway. (**a**) AO staining demonstrated that knockdown of ptgs1 (cox-1), ptgs2a (cox-2a), and ptgs2b (cox-2b) by morpholino did not rescue the apoptotic neurons induced by sulindac in the midbrain of zebrafish embryos. (**b**) Western blot analysis showed that the protein levels of Cox-1 and Cox-2 were significantly decreased after morpholinos (MOs) microinjection. (**c**) The data presented as the means ± SDs (n = 3 independent biological replicates). Statistics calculated by unpaired two-tailed Student's t test. Source data are provided as a Source Data file. β-actin was used as an internal control. (**d**) AO staining demonstrated that prostaglandin E2 (10 μM) did not rescue the apoptotic neurons induced by sulindac in the midbrain of zebrafish embryos. The midbrain is indicated by dashed lines.

Target Key	Target Name	Description	P-Value
GH1_RABIT	PTGS1	Prostaglandin G/H synthase 1	8.18E-19
RXRA_HUMAN	RXRA	Retinoic acid receptor RXR-alpha	7.61E-10
PGH2_RABIT	PTGS2	Prostaglandin G/H synthase 2	6.54E-09
GLRA1_HUMAN	GLRA1	Glycine receptor subunit alpha-1	6.17E-08
PD2R2_HUMAN	PTGDR2	Prostaglandin D2 receptor 2	3.15E-07
PGH1_SHEEP	PTGS1	Prostaglandin G/H synthase 1	1.16E-06
ALDR_RAT	Akr1b1	Aldo-keto reductase family 1 member B1	1.25E-06

Supplementary Fig. 12 (related to Fig. 5) The human RXRa is one of the top-ranked potential targets of sulindac. Results of predicting potential targets for sulindac using the SEA Search Server database(https://sea.bkslab.org/).

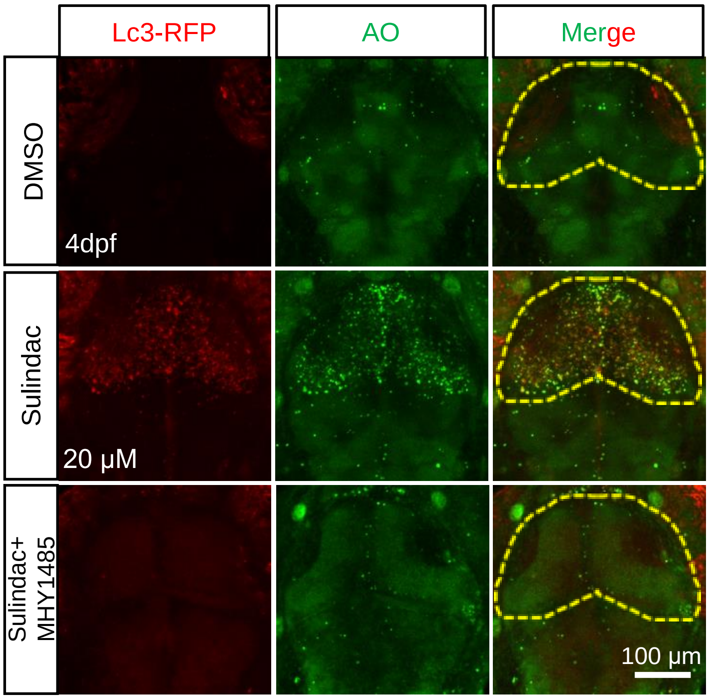

Supplementary Fig. 13 (related to Fig. 5) MHY1485 could rescue sulindac-induced autophagic cell death in zebrafish larvae. Autophagy and apoptosis were evaluated by Lc3-RFP and AO signals when treated with MHY1485 (5 μM) before sulindac treatment. Results showed that MHY1485 (an agonist of mTOR) attenuated the autophagic cell death induced by sulindac in the zebrafish larvae. The experiment was repeated twice with similar results, and three zebrafish were observed in each group.

a

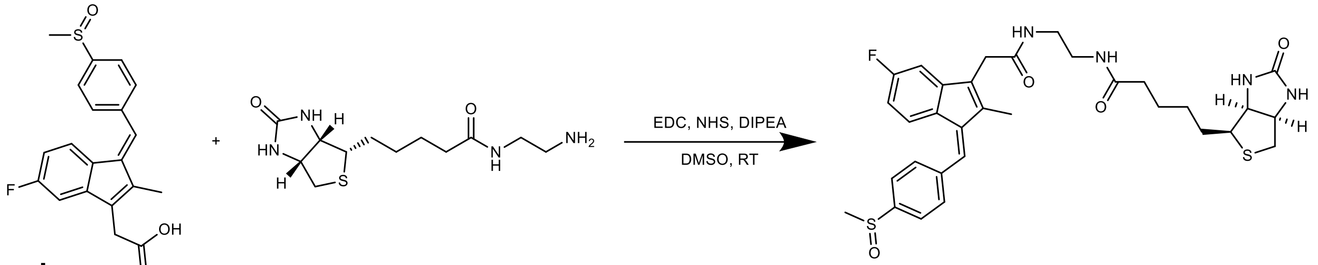
EDC, NHS, DIPEA
DMSO, RT

b

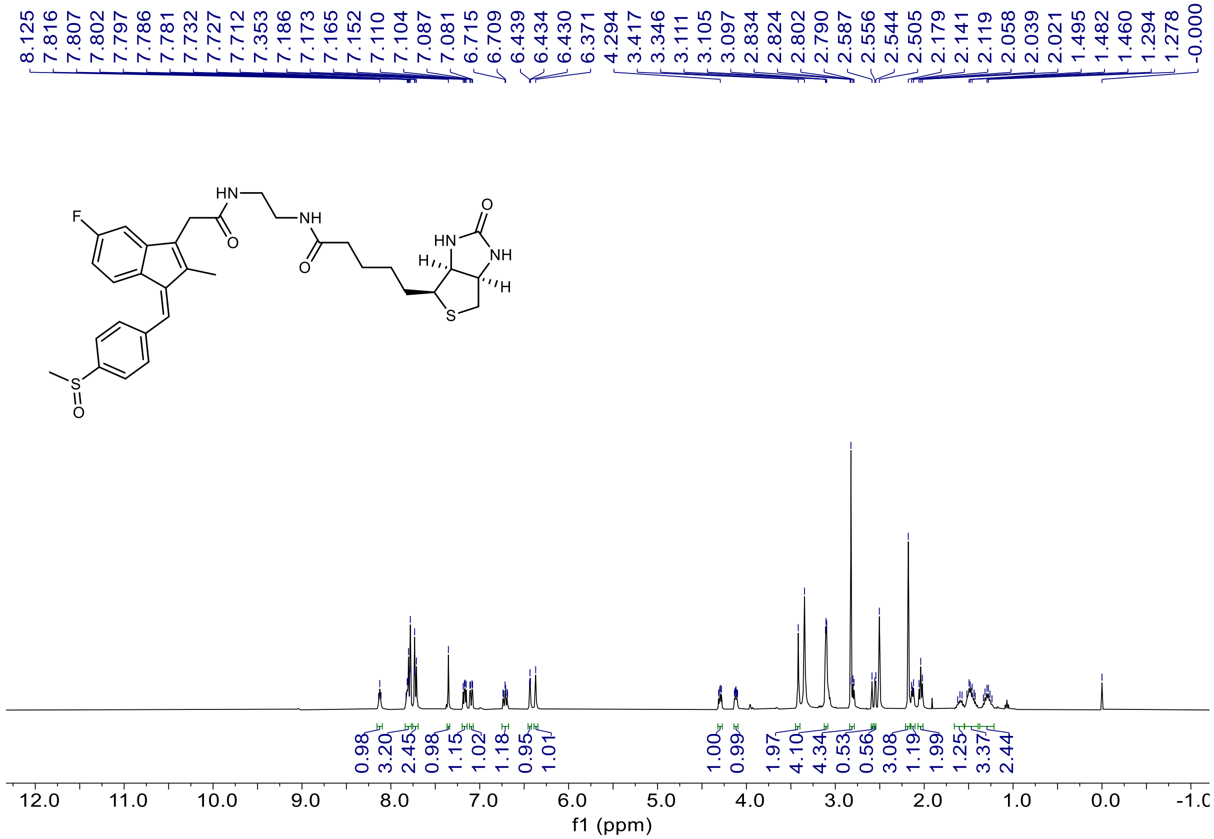
8.125 7.816 7.807 7.802 7.797 7.786 7.781 7.732 7.727 7.712 7.353 7.186 7.173 7.165 7.152 7.110 7.104 7.087 7.081 6.715 6.709 6.439 6.434 6.430 6.371 4.294 3.417 3.346 3.111 3.105 3.097 2.834 2.824 2.802 2.790 2.587 2.556 2.544 2.505 2.179 2.141 2.119 2.058 2.039 2.021 1.495 1.482 1.460 1.294 1.278 -0.000
0.98 3.20 2.45 0.98 1.15 1.02 1.18 0.95 1.01 1.00 0.99 1.97 4.10 4.34 0.53 0.56 3.08 1.19 1.99 1.25 3.37 2.44
f1 (ppm)

c

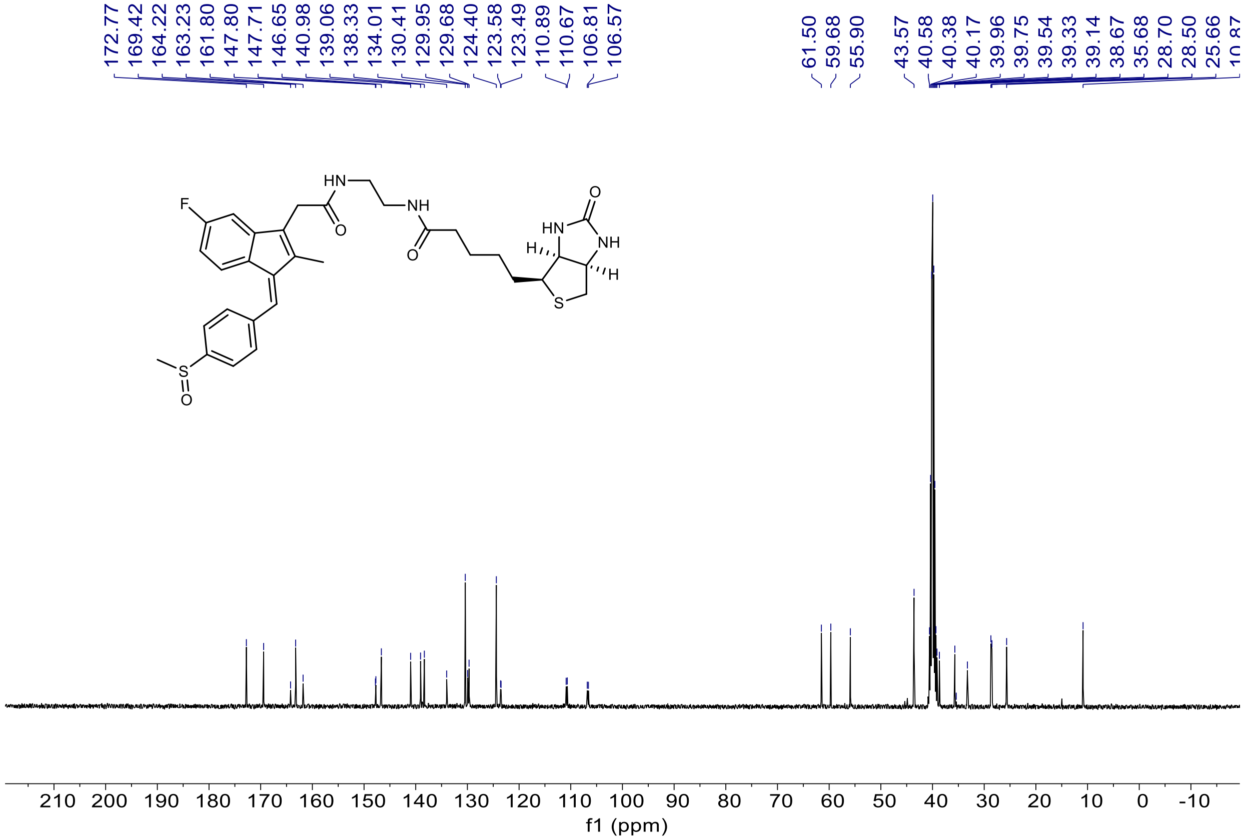
172.77 169.42 164.22 163.23 161.80 147.80 147.71 146.65 140.98 139.06 138.33 134.01 130.41 129.95 129.68 124.40 123.58 123.49 110.89 110.67 106.81 106.57
61.50 59.68 55.90 43.57 40.58 40.38 40.17 39.96 39.75 39.54 39.33 39.14 38.67 35.68 28.70 28.50 25.66
f1 (ppm)

d

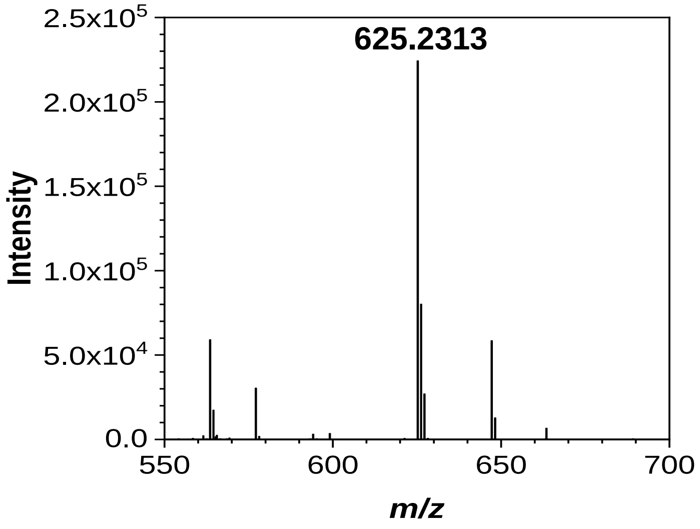

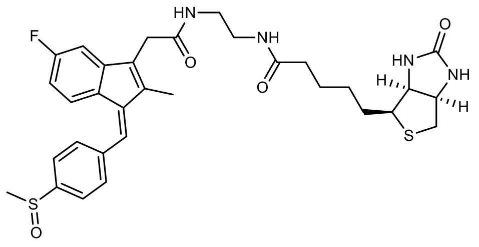

ESI-HRMS: m/z $[M+H]^+$ calcd. for $C_{32}H_{38}FN_4O_4S_2^+$ 625.2240

Supplementary Fig. 14 Synthesize and structure identification of sulindac-Biotion. (**a**) Synthesize routes of sulindac-Biotion. (**b**) ^1H NMR of sulindac-Biotion. (**c**) ^{13}C NMR of sulindac-Biotion. (**d**) Mass spectrometry showed the molecular weight of sulindac-Biotion.

Supplementary Table 1. List of 46 NSAIDs.

MOLENAME	Cas#	Formula	Concentration (μM)	Significance
Meclofenamate Sodium	6385-02-0	$C_{14}H_{10}Cl_2NNaO_2$	50	NO
Fenspiride Hydrochloride	5053/8/7	$C_{15}H_{21}ClN_2O_2$	10	NO
Naproxen (+)	22204-53-1	$C_{14}H_{14}O_3$	20	NO
Oxyphenbutazone	129-20-4	$C_{19}H_{20}N_2O_3$	10	NO
Phenylbutazone	50-33-9	$C_{19}H_{20}N_2O_2$	5	NO
Fenoprofen6	31879-05-7	$C_{15}H_{14}O_3$	20	NO
Flufenamic Acid	530-78-9	$C_{14}H_{10}F_3NO_2$	20	NO
Mefenamic Acid	61-68-7	$C_{15}H_{15}NO_2$	1	NO
Ketoprofen	22071-15-4	$C_{16}H_{14}O_3$	10	NO
Flunixin Meglumine	42461-84-7	$C_{21}H_{28}F_3N_3O_7$	1	NO
Carprofen	53716-49-7	$C_{15}H_{12}ClNO_2$	20	NO
Dexamethasone Sodium Phosphate	2392-39-4	$C_{22}H_{28}FNa_2O_8P$	50	NO
Felbinac	5728-52-9	$C_{14}H_{12}O_2$	50	NO
Diclofenac Sodium	15307-79-6	$C_{14}H_{10}Cl_2NNaO_2$	20	NO
Diflunisal	22494-42-4	$C_{13}H_8F_2O_3$	20	NO
Flurbiprofen	5104-49-4	$C_{15}H_{13}FO_2$	10	NO
Ibuprofen	15687-27-1	$C_{13}H_{18}O_2$	50	NO
Indomethacin	53-86-1	$C_{19}H_{16}ClNO_4$	5	NO
Indoprofen	31842-01-0	$C_{17}H_{15}NO_3$	5	NO
Lumiracoxib	220991-20-8	$C_{15}H_{13}ClFNO_2$	5	NO
Piroxicam	36322-90-4	$C_{15}H_{13}N_3O_4S$	50	NO
Sulindac	38194-50-2	$C_{20}H_{17}FO_3S$	20	YES
Fenbufen	36330-85-5	$C_{16}H_{14}O_3$	5	NO
Firocoxib	189954-96-9	$C_{17}H_{20}O_5S$	10	NO
Tenoxicam	59804-37-4	$C_{13}H_{11}N_3O_4S_2$	1	NO
Isoxicam	34552-84-6	$C_{14}H_{13}N_3O_5S$	1	NO
Celecoxib	169590-42-5	$C_{17}H_{14}F_3N_3O_2S$	10	NO
Nabumetone	42924-53-8	$C_{15}H_{16}O_2$	10	NO
Ketorolac Tromethamine	74103-07-4	$C_{19}H_{24}N_2O_6$	5	NO
Rofecoxib	162011-90-7	$C_{17}H_{14}O_4S$	20	NO
Meloxicam	71125-38-7	$C_{14}H_{13}N_3O_4S_2$	20	NO
Deracoxib	169590-41-4	$C_{17}H_{14}F_3N_3O_3S$	10	NO
Oxaprozin	21256-18-8	$C_{18}H_{15}NO_3$	5	NO
Dexibuprofen	51146-56-6	$C_{13}H_{18}O_2$	50	NO
Tolmetin	64490-92-2	$C_{15}H_{18}NNaO_5$	20	NO
Benzydamine Hydrochloride	132-69-4	$C_{19}H_{23}N_3O.HCl$	10	NO
Lornoxicam	70374-39-9	$C_{13}H_{10}ClN_3O_4S_2$	5	NO
Zomepirac Sodium	64092-49-5	$C_{15}H_{17}ClNNaO_5$	20	NO
Aspirin	50-78-2	$C_9H_8O_4$	50	NO
Acetaminophen	103-90-2	$C_8H_9NO_2$	50	NO
Zaltoprofen	74711-43-6	$C_{17}H_{14}O_3S$	5	NO
Parecoxib	198470-84-7	$C_{19}H18N_2O_4S$	10	NO
SC-560	188817-13-2	$C_{17}H_{12}ClF_3N_2O$	5	NO
NS-398	123653-11-2	$C_{13}H_{18}N_2O_5S$	5	NO
Etoricoxib	202409-33-4	$C_{18}H_{15}ClN_2O_2S$	10	NO
Nimesulide	51803-78-2	$C_{13}H_{12}N_2O_5S$	5	NO

Supplementary Table 2. List of the annotation of 22 clusters of whole-brain sample.

Culster	Cell type	Abbreviation
0	neurons	neuron
1	precursor	precursor
2	neurons (glutamatergic, midbrain, optic tectum)	m-glu
3	Midbrain/Thalamus	m-th
4	retina neuroblasts (amacrine/horizontal)	r-neuroblasts
5	granule cells	granule
6	midbrain (optic tectum)	m-optic tectum
7	mid-hind boundary (gabaergic)	mhb-gaba
8	radial glia	radial-glia
9	progenitor	progenitor
10	hindbrain/cranial nerve	hindbrain/cranial nerve
11	retina (RGC)	rgc
12	ventral forebrain (gabaergic)	v-f-gaba
13	vagal	vagal
14	ganglion	ganglion
15	retina (photoreceptor precursor cells)	r-pho-pre
16	telencephalon (pallium), glutamatergic	pallium-glu
17	hypothalamus	hyp
18	purkinje neurons	purkinje
19	retina (muller glia)	r-m-glia
20	dorsal habenula	d-habenula
21	cornea	cornea

Supplementary Table 3. List of the maker genes for each cluster definition.

culster	cell type	cell numbers	marker
0	neurons	1614	gpm6ab, rtn1a, tubb5, elavl4, lhx9, fez1, elavl3
1	precursor	1033	tuba8l, her15.1, her15.2, her4.2, her2, mki67, pcna, dla, ccnd1, stmn1a, nusap1, tpx2
2	neurons (gabaergic, midbrain, optic tectum)	828	gad2, slc6a1b, slc32a1, snap25a, elavl4, gad1b, atp6v0cb, stmn2a, atpv0e2, lhx5, tal1, lhx1a, fgf12a
3	Midbrain/Thalamus	671	pou4f2, barhl1a, barhl2, zgc:158291, pou3f1, sox6, pou4f1, lef1, lhx9, lhx2b, tcf7l2,
4	retina neuroblasts (amacrine/horizont al)	554	hes2.2, atoh7, olig2, foxn4, gadd45gb.1, neurod4, cldn5b, ascl1a, neurog1, hes6
5	granule cells	503	zic2a, neurod1, zic5, cspg5b, nebl, zic1, zbtb18, fat2, zic4, gsg1l, draxin, olfm2b
6	midbrain (optic tectum)	496	tal1, zfpm2b, gata3, sox14, lhx1a, gata2a, emx2, scrt1a, uncx, pax7a, lhx5, slc6a1b, tcf7l2, tfap2e, lhx1a, gad2
7	mid-hind boundary (gabaergic)	402	pax2a, gbx1, klhl14, pax8, hoxd3a, snap25a, mab21l2, hoxb3a, elavl4
8	radial glia	375	fabp7a, glula, slc1a2b, her4.1, atp1a1b, atp1b4, cx43, slc1a3b, mfge8a, her4.2, s1pr1
9	progenitor	359	her4.2, her15.1, her15.2, her4.1, her2, dla, her12, hmgb2b, sox19a, sox3, ascl1a, dlb, hmgb2a
10	hindbrain/cranial nerve	353	urp1, hoxb5b, hoxb5a, elavl4, slc6a5, hoxc5a, hoxb6b, hoxc1a, meis3, tmie, hoxb6a
11	retina (RGC)	297	rbpms2b, isl2b, rbpms2a, pou4f2, tkta, pou4f1, irx4a, satb2, oaz2b, pou6f2, pax6a, sncga, grin1a
12	ventral forebrain (gabaergic)	295	dlx5a, dlx2a, gad2, dlx6a, lhx6, vax1, gad1b, dlx1a, islr2, slc32a1, slc6a1b, necab1
13	vagal	198	prph, phox2a, phox2bb, tac1, slc18a3a, neflb, isl2a, slc5a7a, nefmb
14	ganglion	169	serpinf1,twist1a,slc16a9a,six1a,slc12a7b, eya2
15	retina (photoreceptor precursor cells)	161	rs1a, crx, gngt2a, tulp1a, elovl4b, six7, tmem244, otx5, arl13a, opn6a
16	telencephalon (pallium) glutamatergic	154	eomesa, tbr1b, emx3, neurod6a, palmdb, neurod6b, tac3b, slc17a6a, olfm1a, draxin, r3hdm1, bhlhe22, bhlhe23
17	hypothalamus	91	trh, fezf1, scg2b, plk2b, rasd1, rgs5b, bhlhe41, cpne2
18	purkinje neurons	57	aldoca, pvalb7, pcp4l1, dazap1, ca8, casq2, itpr1b, dgkh, grm1a, plxdc1
19	retina (muller glia)	50	ca14, hyal6, vil1, anks4b, gpr37a, ush1c, rlbp1a, glula, fabp7a, glulb, acd7, rhbg
20	dorsal habenula	42	gng8, kctd12.1, cnih3, NWD2, synpr, syt6b, g0s2, bean1, pou4f1 syt9b, zic2a, zic3, zic1, ngb
21	cornea	42	scinla, gyg1b, qdpra, pcbd1, tubb6, pitx2

Supplementary Table 4. List of primary antibodies information for western blot.

Antibodies	Suppliers	Catalog Number	Concentration	Experiments
Rabbit polyclonal anti-Lc3b	Novus	NB100-2220	1:1000	Western blot
Rabbit monoclonal anti-p-Akt (Ser473)	Cell Signaling Technology	4060S	1:1000	Western blot
Rabbit monoclonal anti-Akt	Cell Signaling Technology	4691S	1:1000	Western blot
Rabbit polyclonal anti-p62	Novus	NBP1-48320	1:2000	Western blot
Rabbit polyclonal anti-Beclin 1	Proteintech	11306-1-AP	1:1000	Western blot
Rabbit polyclonal anti-Caspase-3	Abcam	ab13847	1:500	Western blot
Mouse monoclonal anti-Caspase-9	Cell Signaling Technology	9508S	1:500	Western blot
Rabbit polyclonal anti-Bcl2	Proteintech	12789-I-AP	1:1000	Western blot
Rabbit polyclonal anti-Bax	Proteintech	50599-2-Ig	1:1000	Western blot
Mouse monoclonal anti-Cytochrome c	Santa Cruz	sc-13560	1:200	Western blot
Rabbit monoclonal anti-COVI	Cell Signaling Technology	4850T	1:1000	Western blot
Rabbit polyclonal anti-PARP	Proteintech	166520-1-Ig	1:1000	Western blot
Rabbit polyclonal anti-PI3K	Affinity Biosciences	AF6242	1:1000	Western blot
Rabbit polyclonal anti-p-mTOR (ser2448)	Cell Signaling Technology	2971S	1:1000	Western blot
Rabbit polyclonal anti-mTOR	Cell Signaling Technology	2972S	1:1000	Western blot
Rabbit polyclonal anti-GAD67	Abcam	ab97739	1:1000	Western blot
Rabbit polyclonal anti-Akt1	Invitrogen	PA5-29169	1:500	Western blot
Mouse monoclonal anti-p-Akt1 (Ser473)	Invitrogen	MA1-20325	1:500	Western blot
Mouse monoclonal anti-Akt2	Santa Cruz	sc-81436	1:500	Western blot
Rabbit monoclonal anti-p-Akt2 (Ser474)	Cell Signaling Technology	8599S	1:500	Western blot
Rabbit polyclonal anti-Akt3	Proteintech	21641-1-AP	1:500	Western blot
Rabbit polyclonal anti-p-Akt3 (Ser472)	Invitrogen	PA5-12898	1:500	Western blot
Mouse monoclonal anti-RXRα	Santa Cruz	sc-541091	1:200	Western blot
Rabbit polyclonal anti-Cox-1	Proteintech	13393-1-AP	1:500	Western blot
Rabbit polyclonal anti-Cox-2	Proteintech	12375-1-AP	1:500	Western blot
Mouse monoclonal anti-GAPDH	Proteintech	60004-1-Ig	1:2000	Western blot
Mouse monoclonal anti-β-actin	Proteintech	66009-1-Ig	1:2000	Western blot
