# Supplementary material for: Sulindac selectively induces autophagic apoptosis of GABAergic neurons and alters motor behaviour in zebrafish
Source: Nat Commun. 2023 Sep 2;14:5351. doi: 10.1038/s41467-023-41114-y (PMC10475106; doi:10.1038/s41467-023-41114-y)
Supplement: Supplementary file 2 — Reporting Summary [file 41467_2023_41114_MOESM2_ESM.pdf]

nature portfolio

Corresponding author(s): Zhibin Haung, Wenqing Zhang, Juan Shen

Last updated by author(s): Aug 10, 2023

Reporting Summary

Nature Portfolio wishes to improve the reproducibility of the work that we publish. This form provides structure for consistency and transparency in reporting. For further information on Nature Portfolio policies, see our Editorial Policies and the Editorial Policy Checklist.

Statistics

For all statistical analyses, confirm that the following items are present in the figure legend, table legend, main text, or Methods section.

n/a	Confirmed	
☐	☒	The exact sample size (*n*) for each experimental group/condition, given as a discrete number and unit of measurement
☐	☒	A statement on whether measurements were taken from distinct samples or whether the same sample was measured repeatedly
☐	☒	The statistical test(s) used AND whether they are one- or two-sided<br>*Only common tests should be described solely by name; describe more complex techniques in the Methods section.*
☐	☒	A description of all covariates tested
☐	☒	A description of any assumptions or corrections, such as tests of normality and adjustment for multiple comparisons
☐	☒	A full description of the statistical parameters including central tendency (e.g. means) or other basic estimates (e.g. regression coefficient) AND variation (e.g. standard deviation) or associated estimates of uncertainty (e.g. confidence intervals)
☐	☒	For null hypothesis testing, the test statistic (e.g. *F*, *t*, *r*) with confidence intervals, effect sizes, degrees of freedom and *P* value noted<br>*Give P values as exact values whenever suitable.*
☒	☐	For Bayesian analysis, information on the choice of priors and Markov chain Monte Carlo settings
☒	☐	For hierarchical and complex designs, identification of the appropriate level for tests and full reporting of outcomes
☒	☐	Estimates of effect sizes (e.g. Cohen's *d*, Pearson's *r*), indicating how they were calculated

Our web collection on statistics for biologists contains articles on many of the points above.

Software and code

Policy information about availability of computer code

Data collection	movement behavior EthoVision XT 15 (zebrafish); Tracking Master V4.10 (mice)<br>Western blot:the MiniChemi image system<br>FACS:Beckman Coulter, USA<br>Confocal imaging:Zeiss 800 confocal microscope
Data analysis	All the statistical analyses were performed in GraphPad Prism (version 8) and ImageJ software (version 1.53k); Cell Ranger (v5.0) and Seurat (v3.1.1) were used to analyze single cell data.

For manuscripts utilizing custom algorithms or software that are central to the research but not yet described in published literature, software must be made available to editors and reviewers. We strongly encourage code deposition in a community repository (e.g. GitHub). See the Nature Portfolio guidelines for submitting code & software for further information.

Data

Policy information about availability of data

All manuscripts must include a data availability statement. This statement should provide the following information, where applicable:

- Accession codes, unique identifiers, or web links for publicly available datasets
- A description of any restrictions on data availability
- For clinical datasets or third party data, please ensure that the statement adheres to our policy

The single-cell RNA-sequencing data from the whole brains of zebrafish larvae had been deposited to the GEO database by the accession number GSE212888: Go to https://www.ncbi.nlm.nih.gov/geo/query/acc.cgi?acc=GSE212888. Additional primary data are available in the article or the online supplementary Data. Source data are provided with this paper.

Human research participants

Policy information about studies involving human research participants and Sex and Gender in Research.

Reporting on sex and gender	N/A
Population characteristics	N/A
Recruitment	N/A
Ethics oversight	N/A

Note that full information on the approval of the study protocol must also be provided in the manuscript.

Field-specific reporting

Please select the one below that is the best fit for your research. If you are not sure, read the appropriate sections before making your selection.

☒ Life sciences ☐ Behavioural & social sciences ☐ Ecological, evolutionary & environmental sciences

For a reference copy of the document with all sections, see nature.com/documents/nr-reporting-summary-flat.pdf

Life sciences study design

All studies must disclose on these points even when the disclosure is negative.

Sample size	Sample sizes were not predetermined based on statistical methods and the exact sample sizes (n) used to calculate statistics are provided in the figure legends. Sample sizes were chosen based an previous publications and experiment types and are indicated in each figure legend. (DOI: 10.1182/blood-2015-12-686147; DOI: 10.1161/CIRCULATIONAHA.121.055468; DOI: 10.1038/s41467-022-35540-7; DOI: 10.1038/nature14325.
Data exclusions	No data was excluded from the analyses.
Replication	Reported results were consistently replicated across multiple experiments where all replicates generating similar results. The number of experiments is specified in each Figure caption.
Randomization	The allocation of zebrafish or mice samples were random, and all mice were age- and sex-matched.
Blinding	Blinding during collection was not needed because conditions were well controlled. Blinding during analysis was not feasible because the results were quantitative and did not require subjective judgment or interpretation. Blinding is not typically used in the field.

Reporting for specific materials, systems and methods

We require information from authors about some types of materials, experimental systems and methods used in many studies. Here, indicate whether each material, system or method listed is relevant to your study. If you are not sure if a list item applies to your research, read the appropriate section before selecting a response.

Materials & experimental systems

n/a	Involved in the study
☐	☒ Antibodies
☒	☐ Eukaryotic cell lines
☒	☐ Palaeontology and archaeology
☐	☒ Animals and other organisms
☒	☐ Clinical data
☒	☐ Dual use research of concern

Methods

n/a	Involved in the study
☒	☐ ChIP-seq
☐	☒ Flow cytometry
☒	☐ MRI-based neuroimaging

Antibodies

Antibodies used

Following antibodies were used: Lc3b (Novus, #NB100-2220, 1:1000), p-AKT (Ser473, CST, #4060, 1:1000), AKT (CST, #4691, 1:1000), p62 (Novus, NBP1-48320, 1:2000), Beclin1 (Proteintech, #11306-1-AP, 1:1000), caspase-3 (Abcam, #ab13847, 1:500), caspase-9 (CST, #9508s, 1:500), Bcl2 (Proteintech, #12789-I-AP, 1:1000), Bax (Proteintech, #50599-2-Ig, 1:1000), cytochrome c (Santa Cruz, #sc-13560, 1:200), COVI (CST, #4850T 1:1000), PARP (Proteintech, #166520-1-lg, 1:1000), PI3K (Affinity Biosciences, #AF6242, 1:1000), p-mTOR (ser2448, CST, #2971, 1:1000), mTOR (CST, #2972, 1:1000), GAD1/GAD67 (Abcam, #ab97739,1:1000), Akt1 (Invitrogen, PA5-29169, 1:500), p-Akt1 (Invitrogen, MA1-20325, 1:500), Akt2 (Santa Cruz, sc-81436, 1:500), p-Akt2 (Ser474, CST, #8599S, 1:500), Akt3 (Proteintech, 21641-1-AP, 1:500), p-Akt3 (Ser472, Invitrogen, PA5-12898, 1:500), RxRα (Santa Cruz, sc-541091, 1:200), Cox-1 (Proteintech, 13393-1-AP, 1:500), Cox-2 (Proteintech, 12375-1-AP, 1:500), mCherry (Abcam, #ab125096, 1:500), Alexa Fluor 488-conjugated anti-rabbit (Invitrogen, #A32731, 1:400), Alexa Fluor 555-conjugated anti-mouse (Invitrogen, #A32727, 1:400), GAPDH (Proteintech, #60004-1-Ig, 1:2000) and actin (Proteintech, #66009-1-Ig, 1:2000).

Validation

All the primary antibodies used in this study are very common and have been used extensively in studies, and all antibodies have been reported for use in zebrafish. In addition, all antibodies were validated by the supplier. All respective validation data are available on the manufacturer's website and reported articles.

Lc3b: validated in: Díaz-Casado M E, Rusanova I, Aranda P, et al. In vivo determination of mitochondrial respiration in 1-methyl-4-phenyl-1, 2, 3, 6-tetrahydropyridine-treated zebrafish reveals the efficacy of melatonin in restoring mitochondrial normalcy[J]. Zebrafish, 2018, 15(1): 15-26.

p-AKT: validated in: Zhang T, Alonzo I, Stubben C, et al. A zebrafish model of Combined Saposin Deficiency identifies acid sphingomyelinase as a potential therapeutic target[J]. Disease Models & Mechanisms, 2023: dmm. 049995.

AKT: validated in: Zhang T, Alonzo I, Stubben C, et al. A zebrafish model of Combined Saposin Deficiency identifies acid sphingomyelinase as a potential therapeutic target[J]. Disease Models & Mechanisms, 2023: dmm. 049995.

P62: validated in: Kim S H, Cho Y S, Kim Y, et al. Endolysosomal impairment by binding of amyloid beta or MAPT/Tau to V-ATPase and rescue via the HYAL-CD44 axis in Alzheimer disease[J]. Autophagy, 2023: 1-20.

Beclin 1: validated in: Watchon M, Yuan K C, Mackovski N, et al. Calpain inhibition is protective in machado–joseph disease zebrafish due to induction of autophagy[J]. Journal of Neuroscience, 2017, 37(32): 7782-7794.

caspase-3: validated in: Di Paola D, Abbate J M, Iaria C, et al. Environmental Risk Assessment of Dexamethasone Sodium Phosphate and Tocilizumab Mixture in Zebrafish Early Life Stage (Danio rerio)[J]. Toxics, 2022, 10(6): 279.

caspase-9: validated in: Chen K, Li X, Song G, et al. Deficiency in the membrane protein Tmbim3a/Grinaa initiates cold-induced ER stress and cell death by activating an intrinsic apoptotic pathway in zebrafish[J]. Journal of Biological Chemistry, 2019, 294(30): 11445-11457.

Bcl2: validated in: Sun C C, Zhou Z Q, Chen Z L, et al. Identification of potentially related genes and mechanisms involved in skeletal muscle atrophy induced by excessive exercise in zebrafish[J]. Biology, 2021, 10(8): 761.

Bax: validated in: Zhang J, Cui X, Wang L, et al. The mitochondrial thioredoxin is required for liver development in zebrafish[J]. Current Molecular Medicine, 2014, 14(6): 772-782.

cytochrome c: validated in: Zhao X, Ren X, Zhu R, et al. Zinc oxide nanoparticles induce oxidative DNA damage and ROS-triggered mitochondria-mediated apoptosis in zebrafish embryos[J]. Aquatic Toxicology, 2016, 180: 56-70.

COVI: validated in: Yang R M, Tao J, Zhan M, et al. TAMM41 is required for heart valve differentiation via regulation of PINK-PARK2 dependent mitophagy[J]. Cell Death & Differentiation, 2019, 26(11): 2430-2446.

PARP: validated in: Zhao L, Tan J, Li D, et al. SLC39A6/ZIP6 is essential for zinc homeostasis and T-cell development in zebrafish[J]. Biochemical and biophysical research communications, 2019, 511(4): 896-902.

PI3K: validated in: Liao L, Zhou M, Wang J, et al. Identification of the antithrombotic mechanism of leonurine in adrenalin hydrochloride-induced thrombosis in zebrafish via regulating oxidative stress and coagulation cascade[J]. Frontiers in Pharmacology, 2021, 12: 742954.

p-mTOR: validated in: Santos-Ledo A, Garcia-Macia M, Campbell P D, et al. Kinesin-1 promotes chondrocyte maintenance during skeletal morphogenesis[J]. PLoS Genetics, 2017, 13(7): e1006918.

mTOR: validated in: Santos-Ledo A, Garcia-Macia M, Campbell P D, et al. Kinesin-1 promotes chondrocyte maintenance during skeletal morphogenesis[J]. PLoS Genetics, 2017, 13(7): e1006918.

GAD1/GAD67: validated in: Moraga A, Pradillo J M, García-Culebras A, et al. Aging increases microglial proliferation, delays cell migration, and decreases cortical neurogenesis after focal cerebral ischemia[J]. Journal of Neuroinflammation, 2015, 12(1): 1-12.

Akt1: validated in: Chia K, Mazzolini J, Mione M, et al. Tumor initiating cells induce Cxcr4-mediated infiltration of pro-tumoral macrophages into the brain[J]. Elife, 2018, 7: e31918.

p-Akt1: validated in: https://www.thermofisher.cn/cn/zh/antibody/product/Phospho-AKT1-Ser473-Antibody-clone-104A282-Monoclonal/MA1-20325

Akt2: validated in: Halon-Golabek M, Borkowska A, Kaczor J J, et al. hmSOD1 gene mutation-induced disturbance in iron metabolism is mediated by impairment of Akt signalling pathway[J]. Journal of Cachexia, Sarcopenia and Muscle, 2018, 9(3): 557-569.

p-Akt2: validated in: Kumar B, Prasad M, Bhat-Nakshatri P, et al. Normal breast-derived epithelial cells with luminal and intrinsic subtype-enriched gene expression document interindividual differences in their differentiation cascade[J]. Cancer research, 2018, 78(17): 5107-5123.

Akt3: validated in: Yang Y, Wu F, Zhang J, et al. EGR1 interacts with DNMT3L to inhibit the transcription of miR-195 and plays an anti-apoptotic role in the development of gastric cancer[J]. Journal of Cellular and Molecular Medicine, 2019, 23(11): 7372-7381.

p-Akt3: validated in: Quambusch L, Depta L, Landel I, et al. Cellular model system to dissect the isoform-selectivity of Akt inhibitors[J]. Nature Communications, 2021, 12(1): 5297.

RxRα: validated in: Zhou H, Liu W, Su Y, et al. NSAID sulindac and its analog bind RXRα and inhibit RXRα-dependent AKT signaling[J]. Cancer cell, 2010, 17(6): 560-573.

Cox-1: validated in: Sun Q, Shi L, Li S, et al. PET117 assembly factor stabilizes translation activator TACO1 thereby upregulates mitochondria-encoded cytochrome C oxidase 1 synthesis[J]. Free Radical Biology and Medicine, 2023.

Cox-2: validated in: Cao P, Zhang H, Meng H, et al. Celecoxib exerts a therapeutic effect against demyelination by improving the immune and inflammatory microenvironments[J]. Journal of inflammation research, 2020: 1043-1055.

mCherry: validated in: Travnickova J, Muise S, Wojciechowska S, et al. Fate mapping melanoma persister cells through regression and into recurrent disease in adult zebrafish[J]. Disease Models & Mechanisms, 2022, 15(9): dmm049566.

GAPDH: validated in: Truong M E, Bilekova S, Choksi S P, et al. Vertebrate cells differentially interpret ciliary and extraciliary cAMP[J]. Cell, 2021, 184(11): 2911-2926. e18.

actin: validated in: Truong M E, Bilekova S, Choksi S P, et al. Vertebrate cells differentially interpret ciliary and extraciliary cAMP[J]. Cell, 2021, 184(11): 2911-2926. e18.

Animals and other research organisms

Policy information about studies involving animals; ARRIVE guidelines recommended for reporting animal research, and Sex and Gender in Research

Laboratory animals	4-week-old Kunming mice (KM) was used to behavior experiment. Tuebingen strain of zebrafish was used to obtain wild-type (WT) embryos for drug screening. All mice were housed in a facility with a light cycle running from 06:00 to 18:00, and the temperature was maintained at 20–22°C with humidity at 50%. Food and water were available ad libitum.
Wild animals	This study did not involve wild animals.
Reporting on sex	Sex was considered in the study design. KM mice: Half male and half female, Zebrafish embryos: No sex difference.
Field-collected samples	No field-collected samples were used in the present study.
Ethics oversight	All the animal experiments in this study were performed in strict accordance with the institutionally approved protocol according to the Institutional Animal Care and Use Committee of the South China University of Technology. (Ethical approval numbe: 2019079)

Note that full information on the approval of the study protocol must also be provided in the manuscript.

Flow Cytometry

Plots

Confirm that:

☐ The axis labels state the marker and fluorochrome used (e.g. CD4-FITC).

☐ The axis scales are clearly visible. Include numbers along axes only for bottom left plot of group (a 'group' is an analysis of identical markers).

☐ All plots are contour plots with outliers or pseudocolor plots.

☒ A numerical value for number of cells or percentage (with statistics) is provided.

Methodology

Sample preparation	the whole-brain tissue was separated from zebrafish juveniles, and the cell suspension was prepared according to a previously described method(Zhang H, Wang H, Shen X, et al. The landscape of regulatory genes in brain-wide neuronal phenotypes of a vertebrate brain[J]. Elife, 2021, 10: e68224.)
Instrument	Beckman Coulter
Software	FlowJo
Cell population abundance	In normal cells, when the membrane potential is normal, JC-1 enters the mitochondria through the mitochondrial membrane J and forms a polymer that emits red fluorescence due to increased concentration. In apoptotic cells, mitochondrial transmembrane potential is depolarized, JC-1 is released from the mitochondria, the concentration is reduced, and the monomer form that emits green fluorescence is reversed. Therefore, green and red fluorescence can be detected by flow cytometry to qualitatively (cell population shift) and quantitatively (cell population fluorescence intensity) detect changes in mitochondrial membrane potential.
Gating strategy	We use the unstained control group and the stained positive control as a reference to set the preliminary FSC/SSC gates.

☒ Tick this box to confirm that a figure exemplifying the gating strategy is provided in the Supplementary Information.
